# Transcriptome-Wide Analysis of Nitrogen-Regulated Genes in Tea Plant (*Camellia sinensis* L. O. Kuntze) and Characterization of Amino Acid Transporter *CsCAT9.1*

**DOI:** 10.3390/plants9091218

**Published:** 2020-09-17

**Authors:** Xinwan Zhang, Hongling Liu, Elizabeth Pilon-Smits, Wei Huang, Pu Wang, Mingle Wang, Fei Guo, Yu Wang, Ruiyuan Li, Hua Zhao, Dejiang Ni

**Affiliations:** 1 Key Laboratory of Horticultural Plant Biology of Ministry of Education, Huazhong Agricultural University, Wuhan 430070, China; zhangxinwan@webmail.hzau.edu.cn (X.Z.); liuhongling@webmail.hzau.edu.cn (H.L.); huangwtea@webmail.hzau.edu.cn (W.H.); pwang@mail.hzau.edu.cn (P.W.); wangmingle@mail.hzau.edu.cn (M.W.); guofei@mail.hzau.edu.cn (F.G.); catea37@mail.hzau.edu.cn (Y.W.); nidj@mail.hzau.edu.cn (D.N.); 2 College of Horticulture & Forestry Sciences, Huazhong Agricultural University, Wuhan 430070, China; 3 Department of Biology, Colorado State University, Fort Collins, CO 80521, USA; Elizabeth.Pilon-Smits@ColoState.EDU; 4 Key Laboratory of information and computing science Guizhou Province, Guizhou Normal University, Guiyang 550001, China; ruiyuan_li@126.com

**Keywords:** assimilation, *Camellia sinensis*, nitrogen, transport, transcriptome

## Abstract

The vigor of tea plants (*Camellia sinensis*) and tea quality are strongly influenced by the abundance and forms of nitrogen, principally NO_3_^−^, NH_4_^+^, and amino acids. Mechanisms to access different nitrogen sources and the regulatory cues remain largely elusive in tea plants. A transcriptome analysis was performed to categorize differentially expressed genes (DEGs) in roots and young leaves during the early response to four nitrogen treatments. Relative to the continuously nitrogen-replete control, the three nitrogen-deprived and resupplied treatments shared 237 DEGs in the shoots and 21 DEGs in the root. Gene-ontology characterization revealed that transcripts encoding genes predicted to participate in nitrogen uptake, assimilation, and translocation were among the most differentially expressed after exposure to the different nitrogen regimes. Because of its high transcript level regardless of nitrogen condition, a putative amino acid transporter, TEA020444/*CsCAT9.1*, was further characterized in *Arabidopsis* and found to mediate the acquisition of a broad spectrum of amino acids, suggesting a role in amino acid uptake, transport, and deposition in sinks as an internal reservoir. Our results enhance our understanding of nitrogen-regulated transcript level patterns in tea plants and pinpoint candidate genes that function in nitrogen transport and metabolism, allowing tea plants to adjust to variable nitrogen environments.

## 1. Introduction

Increasing agricultural production has been achieved mainly by the application of a dramatic increase of nitrogen (N)-containing fertilizers, as well as by the breeding of high-yielding crops along with improved agronomic practices [1,2,3]. In the past decades, there has been an up to a 20-fold global increase in N fertilizer application [4] and this is expected to further increase by at least three-fold by 2050 [5]. The consequences of increased input of N fertilizers have heightened awareness of the serious concerns of environmental damage to the soil and to water pollution [6]. Thus, the necessity of lowering fertilizer requirement and better N use efficiency (NUE) breeding has been recognized and strengthened as long-standing goals within the plant production sector [7,8,9]. Plants take up N not only as inorganic ions that are reduced to amino acids in roots or shoots, but also as organic forms, and the preference greatly depends on plant species and amino acid concentrations [10,11]. While inorganic-N forms are predominant in most agricultural soils, organic-N forms dominate in specific habitats such as forest, meadows, and organic agricultural ecosystems [12]. Moreover, it was previously shown that amino acid-N is vital to plants even with nitrate and ammonium supply [13].

Tea plant (*Camellia sinensis* L.) is a perennial woody evergreen. Young tea leaves are plucked multiple times a year, and therefore tea plants have a great demand for N. This demand is especially high for green tea and oolong tea production, to achieve a high level of free amino acids (AAs) and nitrogenous aromatic substances. Previous studies have shown that the tea plant prefers ammonium (NH_4_^+^) rather than nitrate (NO_3_^−^) at the N uptake step [14,15,16]. Consequently, N application levels and forms are closely related to tea yield and quality [17,18]. In agricultural practices, the production of tea leaves is tightly linked with the application of fertilizers, and usually, a high amount of compound fertilizer is applied in early winter and nitrogenous fertilizer in spring to achieve optimal yield. However, low NUE is typically observed in agriculture due to N-loss, leaching, and ammonia volatilization [19]. Therefore, it is a major goal of breeding tea varieties with a lower need for N fertilizer [8].

NUE in tea plant is both important and complex because of its special cropping system. It includes aspects of N uptake efficiency, utilization efficiency, and translocation/remobilization efficiency, as well as agronomic aspects [20,21]. A mechanistic understanding of how tea plants respond to N is critical to refine strategies to improve NUE [22,23]. To date, little is known about transcriptome-wide gene expression responses in tea plants under variable N environments. The main objective of this study was to determine differentially expressed genes (DEGs) in tea in response to various N forms and levels by a combined analysis of next-generation sequencing-based RNA-Seq (NGS) and single-molecule real-time (SMRT) sequencing using the PacBio RSII platform. Towards this aim, a transcriptome was identified using PacBio sequencing for a pooled sample of one bud with two young leaves and newly developed white roots from plants grown at different N supplies to capture full-length non-chimeric SMRT cDNA reads. In addition, we produced transcriptome reads using Illumina HiSeq X 10 to identify interesting DEGs regulated by the various N treatments separately. The released tea genome sequence [24] will facilitate characterization of key genes, and will promote germplasm utilization and improvement. Tea Plant Information Archive (TPIA) and SMRT sequence results serve as a relatively reliable reference for the annotation of the tea plant NGS RNA-Seq. It is expected that this study would provide new insights into candidate genes associated with N uptake, translocation, and assimilation in tea plants. These findings are meaningful to guide the further development of N management systems; the optimized N application regimes may be designed to suffice for achieving the attainable yield in different agricultural ecosystems.

In tea leaves, AAs constitute 2.0–6.8% of dry mass and AA level is higher in young leaves [25]. Theanine is a unique AA and it accounts for 50–60% of total free AAs. Additionally, the content and composition of AAs are highly associated with tea aroma and taste [26]. The total AA level varies between tea varieties or cultivars, and with the growth conditions [27,28], cultivation strategies, harvest time and maturity [29,30]. Most AAs are not used for protein biosynthesis and are metabolically inactive; these are either stored in the cytoplasm or in organelles, particularly the vacuole [31]. In the mature tea plant, theanine biosynthesis mostly occurs in roots [32], from where it is transported via the phloem, after loading by transporters, through stems to young buds and leaves. Therefore, amino acid transporters (AATs) in tea plants have received much attention in recent years [33,34,35]. Physiological functions of transporters, mediating N transmembrane transport from the soil into roots, from roots to shoots, and remobilization within the tea plant, should be investigated to highlight the importance of different transport systems and to reveal different AA behavior patterns. In this study, several dozen putative N transporters were identified after exposure of tea plants to different N treatments. These are hypothesized to play key roles in N transport and assimilation, and thus to affect NUE and tea quality. A putative *AAT*, *CsCAT9.1*, was further characterized by stable heterologous expression in *Arabidopsis* and shown to mediate acquisition of a broad spectrum of AAs.

## 2. Results

### 2.1. Transcriptome Analysis Overview Based on PacBio RSII and NGS

To identify transcripts for PacBio RSII, an equal amount of high-quality RNA was pooled from sixteen individual samples of two organ types, bud with two young leaves and newly developed white roots, from the four different N treatments. Multiple size-fractionated libraries (<1 kb, 1–2 kb, 2–3 kb, 3–6 kb) were constructed with 16 SMART cells to avoid loading bias. A total of 156,511 post-filter reads representing 25.3 billion bases were generated by PacBio RSII. In total, 576,255 full-length, non-chimeric reads (flncROI) were obtained as indicated by detection of a distinct poly (A) tail, 5′ and 3′ synthesis primer sequences. After removing the redundant sequences for flncROI using ICE and Quiver software, a total of 204,425 subreads with an average length of 2297 bp were obtained. The sequences were then filtered and corrected to achieve high quality (HQ) consensus sequences with accuracy exceeding 0.99. Furthermore, using a two-step cluster by ICE (Figure S1) and LoRDEC (Figure S2), we finally obtained 49,782 isoforms and 42,923 unigenes.

This study focused on identifying differentially expressed transcripts of early responsive to the N regimes. Totally, 1,002,743 clean reads representing 1972 Gbase were generated (Table S1). The NGS produced reads were mapped using the data generated by PacBio database and the Tea Plant Information Archive (TPIA, http://tpia.teaplant.org) as reference [36].

### 2.2. Unigenes Annotation

The PacBio sequenced isoforms were blasted against the reference database of TPIA for annotation in this study. The result showed that 42,742 isoforms, accounting for 85.86% of total isoforms, were mapped to known genes, and 7040 isoforms were mapped to gene space or introns according to the annotation. All unigenes generated by PacBio were matched against five public databases, NCBI non-redundant (Nr) database, Swiss-prot database (SwissProt), Cluster of Orthologous Groups (COG) of proteins database, Gene Ontology (GO) database, and Kyoto Encyclopedia of Genes and Genomes (KEGG) database by Blast X searches with an e-value cut-off of 1 × 10^−5^. A total of 41,325 (96.28%) unigenes were annotated by BLAST searches against these five databases as previously mentioned (Table 1). In total, 41,238 unigenes were annotated in the Nr database. The species distribution of the best match result was translated from BLASTX results as indicated in Figure S3. The maximum number of BLASTX top hits for best group representatives was found with *Vitis vinifera* (33.62%), followed by *Coffea canephora* (5.14%), *Sesamum indicum* (5.04%), *Theobroma cacao* (4.87%), *Nelumbo nucifera* (4.47%), and others accounted for 46.86%.

To determine functional categories of the PacBio sequenced unigenes, GO term analysis was performed over biological processes, molecular functions, and cellular components. As shown in Figure S4, based on sequence homology, a total of 22,983 (53.54%) unigenes were annotated and categorized into the three clusters of biological processes (12,558, 54.64%), molecular function (20,421, 88.85%), and cellular component (4662, 20.28%). Among biological process, the metabolic process of GO:0008152 and cellular process of GO:0009987 were the most representative terms. Among molecular function categories, most unigenes were annotated in the binding of GO:0005488, catalytic activity of GO:0003824 and transporter activity of GO:0005215. Among cellular component category, cell of GO:0005623 was dominant, followed by cell part of GO:0044464 and membrane GO:0016020.

A total of 26,841 unigenes were annotated by the KOG database and clustered into 26 functional groups (Figure S5). Among them, the largest cluster was ‘general function prediction only’ with 5058 unigenes annotated, followed by ‘signal transduction mechanisms’ with 3111 unigenes annotated. The terms of ‘inorganic ion transport and metabolism’, ‘secondary metabolites biosynthesis, transport and catabolism’, and ‘amino acid transport and metabolism’ were annotated with 872, 908 and 1228 unigenes, respectively.

To predict the possible function of the unigenes in the transcriptome, 18,264 unigenes (42.55%) were annotated by the KEGG database. These unigenes were classified into six categories of cellular processes of transport and catabolism (966 unigenes), environmental information processing of membrane transport and signal transduction (852 unigenes), genetic information processing (3824 unigenes), metabolism (6924 unigenes), and environmental adaptation (547 unigenes) and human diseases (41 unigenes), which were mapped to 274 KEGG pathways (Figure S6). Among these pathways, the ‘carbohydrate metabolism’ comprising 1699 unigenes was dominant, followed by ‘amino acid metabolism’ (1564 unigenes in total), ‘translation’ (1463 unigenes), and ‘folding, sorting and degradation’ (1297 unigenes). Additionally, terms of ‘environmental adaptation’ (547 unigenes) and ‘membrane transport’ (112 unigenes) were indicated and would be implicated in response to different N environments.

### 2.3. Global Analysis of Differentially Expressed Genes (DEGs)

**Figure 1:**
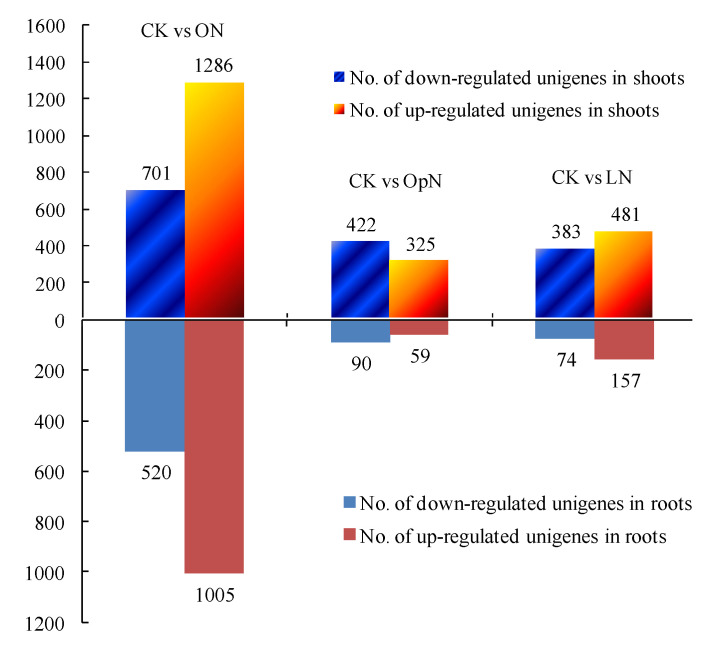
The number of differentially expressed unigenes between the N treatments with the control. The columns above and below the *X*-axis represent the number of DEGs in shoots and in roots, respectively. The numbers of down- and up-regulated unigenes are indicated using blue and orange columns, respectively. To be considered differentially expressed, transcripts must have FPKM > 0.01, 2-fold or greater difference in expression level between treatments, and *p* < 0.05. Note: Seedlings were treated with continuous normal N (CK) as control, and were deprived of N for three days and then resupplied with optimal N (OpN), low N (LN), and organic N (ON) treatments for five hours.

**Figure 2:**
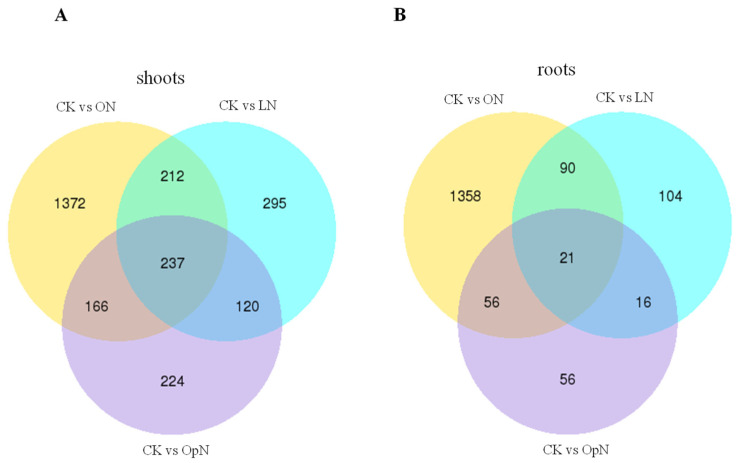
Venn diagrams showing the number of unique and overlapping DEGs for each comparison between N treatments. Compared with the control, the three N treatments shared 237 and 21 DEGs in shoots (**A**) and in roots (**B**), respectively.

In the shoots, a total of 2626 DEGs were regulated by N resupply compared with control (CK): 747 (OpN), 864 (LN) and 1987 (ON) unigenes were identified, respectively, for the three N treatments (Figure 1), and 735 unigenes were detected for multiple N treatments, as shown by the overlap in Venn diagrams (Figure 2). In the roots, 1701 DEGs were observed between N resupply treatments and CK control: 149 (OpN), 231 (LN), 1525 (ON), respectively, with an overlap of 183 unigenes across treatments (Figure 1 and Figure 2).

### 2.4. Transcript Analysis of Genes Involved in N Uptake and Transport

**Figure 3:**
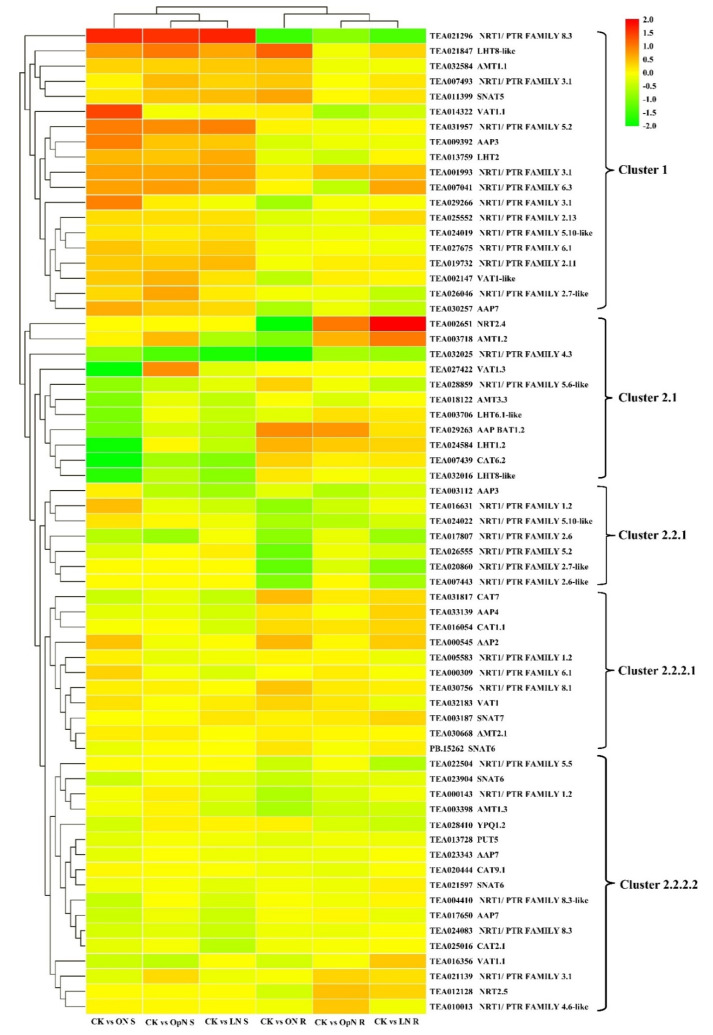
Heat map showing the relative expression of genes involved in N-uptake in roots and N-translocation within tea plant upon the various N treatments, compared with the control. The vertical bar indicates the relative expression ratio, and red, yellow, and green represent transcriptional up-regulation, no change, and down-regulation, respectively.

**Figure 4:**
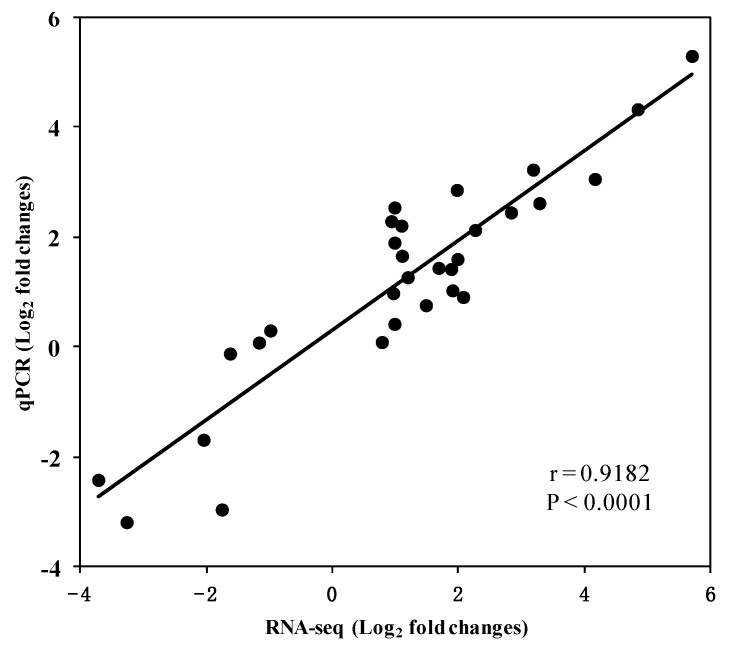
Correlation between RNA-seq and qRT-PCR for the selected ten unigenes. Each point represents a fold change value of expression level in OpN, LN or ON compared with CK in shoots or roots.

The transcript levels of N transporters are of particular interest as they mediate N uptake and translocation within tea plants. The transporters of NRT and AMT family members are responsible for NO_3_^−^ and NH_4_^+^ transport, respectively. The color in Figure 3 shows their fold changes based on the normalized FPKM values between treatments of OpN, LN, ON and the CK control, respectively, in shoots and roots. Figure S7 shows the transcript levels of differentially expressed NRT and AMT transporter genes in shoots and roots, between the three N treatments and CK control. In total, 28 NRT transporters were divided into three major categories in Figure S7, thirteen showing considerable expression levels in shoots and as well in roots, nine mainly and highly expressed in shoots and six in roots, respectively. As shown in Figure 4, eleven *CsNRT1* were up-regulated upon transfer of tea plants to the three N treatments compared with CK in shoots, thus they were grouped in cluster 1. Within cluster 2-1, eight *CsNRT1* transporters showed decreased expression patterns in roots upon the three treatments of N resupply relative to CK. Nine *CsNRT1* genes were slightly induced by three N regimes compared with CK control, both in roots and in shoots. Specifically, those transcripts were abundant under all treatments and probably these candidate genes are responsible for N uptake or translocation, but not necessarily regulated by N regimes.

Ammonium-N has been shown to be preferentially taken up by tea plants. Among genes encoding AMTs, TEA030668/*CsAMT3.1* showed higher expression level in roots than in shoots and similar expression level among the different N treatments. TEA032584/*CsAMT1.1L*’s transcript was somewhat more abundant in the three N treatments compared to CK in shoots, whereas in roots it was only highly regulated by ON. Across the N resupplies, TEA003398/*CsAMT1.3* showed similar transcript levels among the treatments in shoots, while no conspicuous expression was observed in roots. TEA003718/*CsAMT1.2* was more highly expressed by OpN (1.5-fold) and by LN (2-fold), whereas it was less expressed by ON relative to CK in roots; there was almost no expression across the N treatments in shoots. Stable low-level expression and slight decreases of TEA018122/*CsAMT3.3* were observed in shoots across the three N treatments compared to CK, and extremely low expression levels were found in roots.

To some extent, N is taken up in the form of AAs. Assimilated N in the form of AAs are transported throughout the tea plant via the vasculature, loaded by transporters, and these AAs are subsequently accumulated mainly in young buds and developing leaves, either for utilization or storage. As shown in Figure 4, seven AATs were assigned into cluster 1, whose transcripts in shoots were more abundant in the three N treatments compared to CK; in roots, TEA021847/*CsLHT8-Like* showed much higher expression level, especially in ON. Six AATs were grouped together in cluster 2.1. Two LHT family members transporters, *CsLHT8-like* and *CsLHT1.2*, (TEA032016 and TEA024584) presented high expression levels both in roots and in shoots, and in shoots, they showed significantly lower level in ON compared to CK (Figure S8). TEA027422/*CsVAT1.3* showed high transcript levels and they were comparable across different N conditions in the roots; conversely, in the shoots, it showed a strikingly low expression with ON supply. TEA029263/*CsAAP BAT1.2* and TEA007439/*CsCAT6.2* were expressed at a very low level in the shoots, and were at a moderate level and induced by ON in the roots. Seven AATs were grouped together in Cluster 2.2.2.1, and their transcript levels in roots were slightly increased in ON and LN compared with CK. In Cluster 2.2.2.1, all showed much higher expression levels in roots than in shoots, except for TEA016054/*CsCAT1.1* expressing similar levels in shoots and roots. Nine AATs were grouped in Cluster 2.2.2.2 and almost all of them, both in shoots and in roots, showed lower expression in the three treatments relative to CK. The transcript levels of TEA020444, TEA023343, TEA025016, TEA021597 and TEA013728 were abundant in the roots, and TEA028410, TEA013728, TEA023343, TEA021597, TEA017650 and TEA025016 showed high transcript levels in the shoots (Figure S8).

### 2.5. Transcript Analysis for Genes Involved in N Assimilation

Figure S9 showed transcript levels of genes involved in N assimilation. Two *CsNiRs*, TEA000784 and TEA013227, showed higher transcript levels relative to control after the supply of OpN (both almost 2-fold) and LN (3.6- and 2.1-fold, respectively), and lower transcript level than control after supply with ON (0.43- and 0.49-fold respectively) in the roots. In the shoots, the two *CsNiRs* showed slightly increased expression level with the supply of LN and ON, and almost 2-fold with OpN of TEA013227, relative to those of the control. Among the seven *GS* isoforms, TEA032217/*CsGS1.1* had extremely high expression level both in roots and in shoots, and compared to CK in roots, significantly higher in OpN and LN and lower in ON were observed; while in the shoots, higher expression level was only found in LN compared to the control. TEA015198/*CsNodulin/GSL* transcript levels were marginally higher than other *GS* isoforms and significantly lower in the three N treatments relative to CK in the roots, while in the shoots its expression levels were much lower than those in the roots and it showed similar expression across the four N treatments. As for TEA015580/*CsGS1.3*, it showed abundant transcript levels both in shoots and in roots and elevated expression compared with CK in shoots. TEA028194/*CsGS2.1* was expressed at a much higher level in shoots than in roots, whereas TEA032123/*CsCS1.1* was expressed at a slightly higher level in roots than in shoots. Both of TEA034053/*CsCS1.3* and TEA034054/*CsCS1.3* showed sharply lower transcript levels relative to other *CsCSs* members. Three *NADH-GOGAT* genes’ transcript levels were differentially expressed in response to distinct N treatments and tissues, showing significantly higher expression in roots than in shoots. Compared to their corresponding CK controls, TEA026779/*CsNADH-GOGAT1* and TEA003892/*CsNADH-GOGAT1* showed similar root transcript levels in OpN and lower expression in LN and ON, while TEA011569/*CsNADH-GOGAT1* presented higher expression level in OpN and LN and lower in ON. The two *CsFd-GOGAT* genes were expressed at much higher levels in shoots than in roots, and in roots, TEA024031 showed a lower level across N treatments compared to CK. Figure S9 shows the transcript levels of three *CsAlaATs* genes differentially expressed between LN, ON and CK in roots, and a similar expression pattern was observed for TEA023088/*CsAlaAT1* in shoots. Slightly differential expression upon the N treatments were observed for TEA009809/*CsGDH2* and TEA034004/*CsGDH1*, both in shoots and in roots. The transcript level of TEA031206/*CsGDH2* was lower in LN and higher in ON compared with CK in roots, and levels were much lower in shoots. Two *CsGluRs* (TEA024970/*CsGluR3.4-like* and TEA021684/*CsGluR3.2*) were more highly expressed in shoots than in roots, and their transcript levels in the shoots were slightly more expressed in ON than in CK.

### 2.6. RNA Sequencing Validation by qRT-PCR

The validation of the N-responsive transcripts was performed by qRT-PCR analysis with ten unigenes showing differential expression patterns at least across two different N conditions. The biological samples used for RNA-Seq analysis were employed with three replicates. Genes known to be involved in nitrate or ammonium uptake and assimilation, including *NRT2.4*, *NiR*, *NIA*, *AMT1;2*, *GS*, *GLT* homologues, were confirmed by qRT-PCR to exhibit similar expression profiles to those measured by RNA-seq. Additionally, the expression profiles of transcripts annotated as transcription factors, *PIP2.4*, and AATs were measured by qRT-PCR. Expression profiles were expressed as Log_2_ (fold change). The results showed a strong correlation between RNA sequencing and qRT-PCR (*r* = 0.9182, *p* < 0.0001, Figure 4). For each gene, the transcriptome data exhibited a similar expression profile across all N treatments compared with the results from qRT-PCR (Table S2), showing the RNA sequencing results to be reliable.

### 2.7. Subcellular Localization for a Putative Amino Acid Transporter of CsCAT9.1 with Differential Broad-Spectrum for Substrate Selectivity

**Figure 5:**
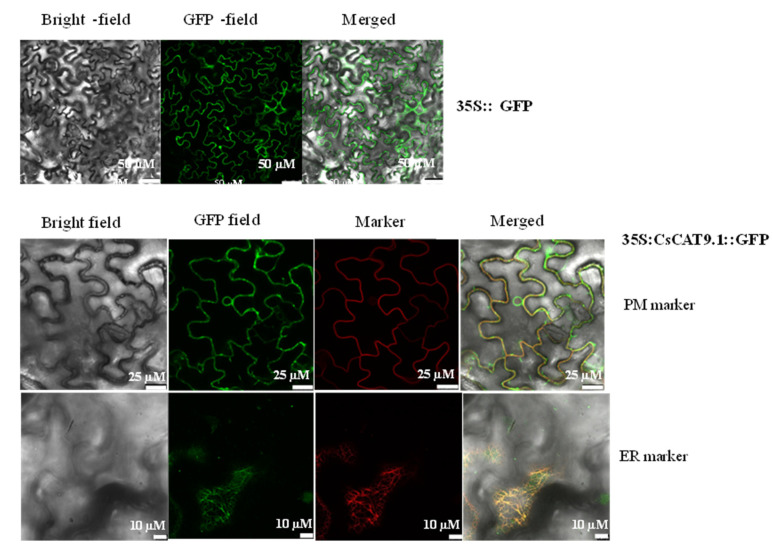
Subcellular localization of transiently expressed fusion proteins for 35S::CsCAT9.1::GFP with plasma membrane marker (PM) and endoplasmic reticulum (ER) marker in *Nicotiana benthamiana* leaves. Scale bars = 50 μM (35S::GFP as control) and 25 μM (35S::CsCAT9.1::GFP) using PM marker, 10 μM (35S::CsCAT9.1::GFP) for ER marker.

In this study, a putative cationic amino acid transporter, *CsCAT9.1*, was found to be stably and highly expressed in the shoots and roots under different N regimes; therefore, it is hypothesized to be an essential candidate for AA transport. To investigate its physiological roles, we cloned it for localization and functional characterization. As shown in Figure 5, the GFP signal of *CsCAT9.1* was co-localized with the fluorescence of plasma membrane marker CBln and endoplasmic reticulum marker mCherry-HDEL.

**Figure 6:**
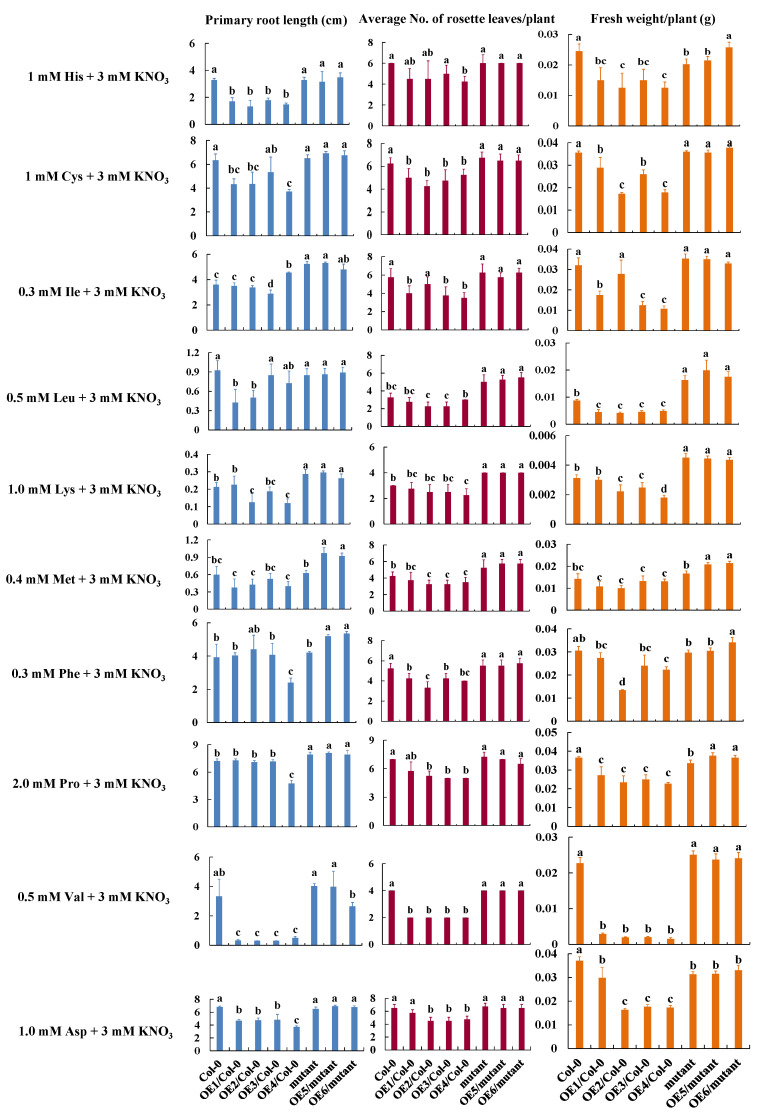
Growth performances of *CsCAT9.1*-OE *Arabidopsis* seedlings grown in medium supplemented with individual amino acids. The NO_3_^−^ treatment served as a control of N source (Figure S10). OE1, OE2, OE3, and OE4 represent independent overexpression lines with WT background, OE5 and OE6 represent independent complementary lines with *cat9* mutant background. Error bars indicate SD. Statistically significant differences are indicated by letters a, b, c, and d (*p* = 0.05).

To figure out AAs substrates for TEA020444/*CsCAT9.1*, *Arabidopsis* plants expressing this gene were grown for ten days on a medium containing 3 mM NO_3_^−^ and individual AAs as N source, in comparison with untransformed plants. Growth performances were quantified for fresh weight, primary root length, and number of rosette leaves. The inhibitory effects of AAs on any detected traits in *CsCAT9.1*-overexpressing (OE) lines might suggest substantial uptake or transport of the corresponding AAs mediated by *CsCAT9.1 in vivo*. As shown in Figure 6, it was observed that the growth of OE lines with *Arabidopsis cat* mutant (AT1G05940) background was comparable with the growth of the WT and the *Arabidopsis cat* mutant, particularly with respect to rosette leaves. The growth inhibition of OE lines in WT background was in sharp contrast to other lines, and the inhibitory effect was likely attributed to the high level of CAT activity due to the overall expression of the two homologs of *CsCAT9.1* and *AtCAT9,* which might indicate the functional overlapping of these two homologs in the transport of these AAs. In all, TEA020444/*CsCAT9.1* expression affected uptake and/or transport the ten AAs summarized in Figure 6. Furthermore, OE lines in the WT background showed severely chlorotic rosette leaves and slightly reduced biomass (NS) in response to separate supplementation with six other AAs: Thea, Gly, Ala, Arg, Gln, and Glu. 

## 3. Discussion

**Figure 7:**
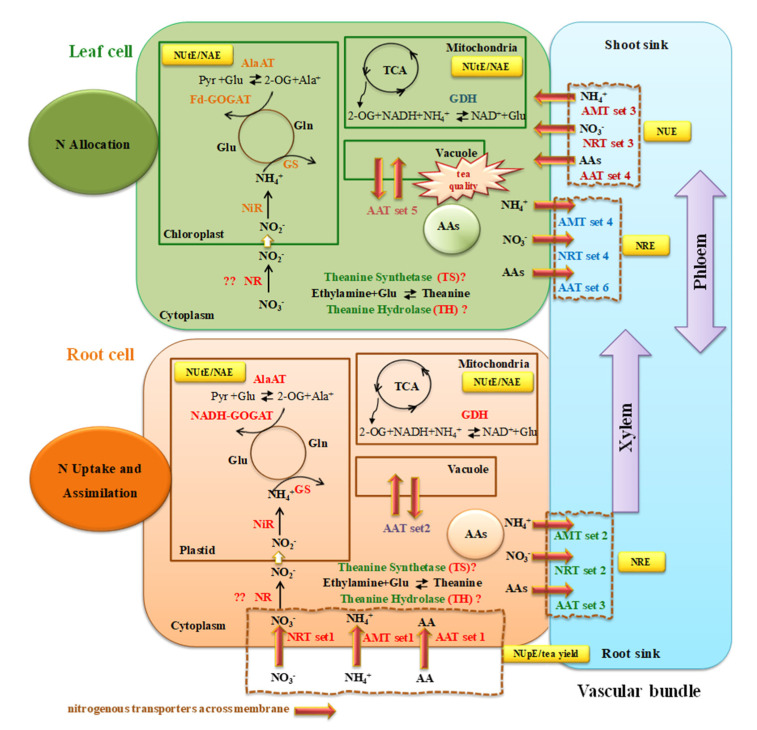
Integrative schematic model of genes proposed to mediate the physiological functions of N uptake, assimilation, and transport (translocation and remobilization) in roots and shoots based on this transcriptome case. Genes in red bold expressed either higher or lower in response to external N treatments.

N is an essential nutrient for tea plants growth and highest content is contained in young harvestable tea shoots mainly in the secondary compounds of caffeine (1,3,7-trimethyl xanthine) and theanine (γ-glutamyl-L-ethylamide), up to 2.80% and 4.53% in young shoots at dry weight [37,38]. Tea plants are characterized by preferential assimilation and uptake of NH_4_^+^ over NO_3_^−^ [15,16]. Therefore, N is one of the most important nutrient determining tea quality and yield. Knowledge of candidate genes genomewide responses to N applications in tea plants is important for the sustainability of the cropping system and economic benefit of higher tea quality and yield. Based on transcriptome comparison of OpN, LN, and ON with the CK control, the potential genes involved in primary N metabolism, including uptake, assimilation and remobilization, were summarized in Figure 7. The complexity of inorganic N uptake in plants involves ammonium and nitrate uptake systems, typically as low-affinity, high-affinity and dual-affinity systems, based on their transport activity [39,40]. In the present study, low-affinity N-related transporters were expected to be induced by OpN resupply, while high-affinity N-related transporters would be up-regulated by LN resupply, and AATs by ON resupply [41,42]. As expected, the elevated or down-regulated expression of various types of N transporter family members were harvested and these transporter genes are supposed to be involved in N uptake and transport within the tea plant (Figure 7 and Figures S7 and S8). The model further hypothesizes that tea plants mediate uptake, translocation, and remobilization of N via these transporters throughout the tea plant, and overall NUE (Figure 7 and Figures S7–S9). Moreover, it may be considered that these transport processes are involved in NUE by regulating transporters and key N assimilation genes’ expression to acclimate to the changing N conditions.

It is known that N starvation can rapidly induce the transcription response of N-related transporters [43], such as *NRT*, and *AMT* families. As expected, a variety of *NRT* members showed a differential response to the N treatments, including 28 *NRT1* family members and two high-affinity transporters, TEA012128/*CsNRT2.5* and TEA002651/*CsNRT2.4*. In roots, the modified steady-state expression pattern partly overlapped between the three N treatments and CK control, especially for TEA002651/*CsNRT2.4* and TEA012128/*CsNRT2.5* being up-regulated in OpN/CK, LN/CK and down-regulated in ON/CK comparisons, suggesting their involvement in N uptake efficiency (NUpE) improvement. Moreover, high transcript level in roots of TEA012128/*CsNRT2.5* with ON supply indicates a role in xylem loading for long-distance transport from roots to shoots. The transcript levels of TEA021296/*CsNRT1* were significantly lower in roots, whereas in shoots they were 3-fold higher with three N treatments compared with the control, implying such a gene may be important for N remobilization in shoots to acclimate to N status. It is possible that TEA020860/*CsNRT1* and TEA017807/NRT1 contribute to N uptake, because they were mainly expressed in the roots and showed decreased expression in response to the LN and ON resupply treatments. The higher expression levels of *CsNRTs* family members TEA029266 and TEA019732 in shoots with ON supply indicates that these genes may be involved in NO_3_^−^ phloem loading or remobilization from shoots to roots, contributing to N remobilization efficiency [20]. The AMT members in the ammonium-preferring tea plant may play important roles, especially in N uptake efficiency. Exposed to the differential N sources, five *CsAMTs* were identified as DEGs and they are implicated either in participating in NH_4_^+^ uptake from the solution or transport between shoots and roots (Figure 7 and Figure S7-4). Transcript levels of TEA003718/*CsAMT1;2* were up-regulated upon LN and OpN resupply, down-regulated in response to ON in the roots, with negligible expression in shoots, suggesting an important role in NH_4_^+^ uptake from medium (Figure S7-4). The constitutively abundant transcript levels of TEA030668/*CsAMT2;1* both in shoots and in roots may suggest important roles in uptake or translocation or remobilization within the tea plant. Taken together, N availability appears to be sensed by tea plants in a form-specific fashion, leading to differences in transcriptome responses; the associated changes in transporter activities likely are pivotal in the plant to adjust to changing N status.

AATs might be involved in the homeostasis of AAs and in their sequestration in organelles; thus, their expression patterns may affect tea taste quality [33,35]. The expression of AATs involved in soil-to-root (uptake) would depend on AA recognition as substrates and AA availability. Therefore, the involvement of AATs in roots consists of three major functions: uptake from the medium, transmembrane transport to target subcellular localization, and xylem loading for long-distance transport. It is intriguing that many of the highly expressed AAT genes in roots and shoots are AAP family members. This implies the importance of AAP transporters in the tea plant, as previously identified [33], as well as in rice [44,45] and *Arabidopsis* [46,47]. This study showed that *CsAAPs* have distinct expression patterns, suggesting functional specialization. Some *CsAAPs* were highly expressed in shoots compared with in roots; therefore, their properties and functions may differ with respect to substrate selectivity and transport activity. The elevated expression of AAT set1 in roots with ON resupply may indicate a function in AA uptake (Figure S11). The high expression of AATs of sets 2 and 3 in roots would explain the transmembrane transport or xylem loading for AAs for further long-distance transport from roots to shoots (Figure S11). On the other hand, AATs with high expression levels in shoots may mediate transmembrane transport for AA homeostasis (AATs set 5), transport from vascular tissues to shoot sinks (AATs set 4), or phloem loading for remobilization from shoots to roots (AATs set 6). Several additional AAT genes showed very low expression levels in both roots and shoots, and might be functionally redundant (Figure S8). Given its stable abundant expression level under different N regimes in the roots and shoots, TEA020444/*CsCAT9.1* is hypothesized to be a major candidate for uptake, translocation, and/or remobilization of AAs, and thus may be involved in NUE.

Uptake of N in roots is regulated by N assimilation and transport within the plant. The higher expression of the two nitrite reductase genes *CsNiRs* (TEA000784 and TEA013227) in roots compared to in shoots indicates that N assimilation is mainly in tea plant roots, which was also reported previously [26]. Owing to the central nature of glutamine synthetase (GS) in N metabolism, changes in GS expression would affect N metabolism and potentially affect NUE. The elevated transcript levels of the genes involved in the N assimilation pathway may give rise to higher N uptake, translocation, yield and better quality, as found in transgenic maize [48] and rice [49] by overexpressing GS. However, comparable transcript levels of GS genes were observed between shoots and roots, except for the isoform TEA028194/*CsGS2.1*. The higher expression of TEA028194/*CsGS2.1* in shoots than in roots might indicate it plays a role in chloroplasts, together with two other GS isoforms, TEA032217/*CsGS1.1* and TEA015580/*CsGS1.3*. The sequence of GS was highly similar to theanine synthetase (TS) [24,50]. TEA015198/*CsNodulinGS* was expressed specifically in the roots and biosynthesis of theanine has been shown to mostly occur in roots [32]. Whether its expression has a physiological consequence on theanine assimilation and accumulation is hard to predict, but it will be an interesting candidate. In mitochondria, GDH is involved in NH_4_^+^ assimilation to catalyse the reversible reaction from NH_4_^+^, NADH and 2-oxoglutarate (2-OG), producing glutamate and NAD^+^. The higher expression of the four *CsGDH* isoforms in roots than in shoots may explain the fact that N assimilation mainly occurs in the roots. The almost 4-fold elevated transcript level of TEA031206/*CsGDH2* upon ON and LN treatments relative to CK in the shoots might indicate that this is an adaptation to low N availability. Slightly higher expression in roots upon ON might be due to the glutamate supplied, as glutamate catalyzes the reversible reaction to 2-oxoglutarate, suggesting GDHs’ important feedback role, affecting the TCA cycle. Therefore, *CsGDHs* will be interesting candidates to further investigate relationships between their expression levels with N assimilation efficiency (NAE) and NUE. The expression levels of three *CsAlaATs* were all higher in roots than in shoots. The studies showed that overexpression of *AlaATs* increases biomass, total N, yield, and key metabolites in rice [51], and maintain yield with much less N application in canola [52]; thus, such a gene would be very promising for genetic engineering of plants with enhanced NUE. Two *CsGluRs* were expressed at higher levels in shoots than in roots, and also showed enhanced expression in response to ON compared to CK in shoots. The reduced growth rate of *AtGluR2*-overexpressing plants has been reported [53]. It is possible that *CsGluRs* contributes to adaptability to the ON condition via a currently unknown mechanism.

The cumulative effect of expression of two homologous *CAT* genes might explain why the *CsCAT9.1* OE lines with WT background showed growth inhibition by the supplied AAs. Assuming comparable transport efficiency, the cumulative effect of *CsCAT9.1* and its homolog *AtCAT9* may be supra-optimal, resulting in growth inhibition in the OE-lines with WT background, not in the *cat* background. In contrast, for amino acid transporter *CsAAP3.1*, the OE lines with *aap3* mutant background and with WT background both showed a serious growth inhibition upon AAs supply (Figure S10). Perhaps *CsAAP3.1* can transport AAs more efficiently than the endogenous *AtAAP3*, resulting in growth inhibition of OE lines with both WT and *aap3* background. Further investigation of transport efficiency and kinetic parameters of *CsCAT9.1* for the candidate AAs is necessary to address these hypotheses.

The transport efficiency of AAs from roots to sink leaves generally determines the tea quality and requires at least two active transport steps for the long-distance transport and partitioning of AAs [54]. This study assessed the response of tea plants to varying N conditions and identified a set of AA transporter candidates (Figure S8). These candidates involved in N transport and assimilation have the potential to increase tea plant NUE and tea quality, and therefore are interesting candidates for further investigation.

## 4. Materials and Methods

### 4.1. Tea Plant Materials and N Treatments

Seedlings (*Camellia sinensis*, cv. Fudingdabai) were cultured in plastic tanks containing nutrient solution with composition as shown in Table S3, and further referred to as CK (N-replete control). After 30 d of pre-growth with refreshment of the solution every 5 d, seedlings developed new white roots. At that point, the plants were exposed to different N treatments. For transcriptome experiments, seedlings of uniform size were divided over the following four N conditions (Table S3): As a continuously N-replete control (CK) treatment, seedlings were treated with 3.34 mM N, consisting of 0.42 mM (NH_4_)_2_SO_4_, 0.42 mM Ca(NO_3_)_2_, and 0.83 mM NH_4_NO_3_. For the other three treatments, the seedlings were deprived of N for three days (other elements stayed constant) and then resupplied for five hours with (I) optimal N (OpN), containing a uniform N composition as CK, (II) low N (LN), consisting of one-tenth level of N as in OpN treatment, and (III) organic N (ON), consisting of theanine, glutamate, glycine, histidine, and valine (predominant composition at ecologically relevant levels in tea garden soil solution), each at 30 μM. The ON treatment was established according to the presence and level of main AAs in the soil solution of a tea garden. For LN and ON treatments, (NH_4_)_2_SO_4_ and Ca(NO_3_)_2_.4H_2_O were replaced by CaSO_4_; all other nutrient concentrations except N in the ON solution were as described for CK. These N treatments were chosen because upon N resupply after deprivation, some specific low-affinity N-related transporters were expected to be induced by OpN resupply, while high-affinity N-related transporters would be up-regulated by LN resupply, and AATs by ON resupply. Each treatment was repeated three times in separate culture tanks, each containing 35 plants. After 5 h of N resupply, samples, newly developed white roots and one bud with the two uppermost young leaves, were separately collected at the same time and immediately frozen in liquid nitrogen and stored at −80 °C for RNA extraction.

### 4.2. RNA Extraction, Next Generation Sequencing, SMRT Sequencing and Differential Expression Analysis

Total RNA of each sample was extracted using the RNeasy plant mini kit (Qiagen China Co., Ltd., Shanghai, China) according to the manufacturer’s instructions. RNA quality was determined with the 2100 Bioanalyzer (Agilent Technologies, Santa Clara, CA, USA) for 28S/18S and RNA integrity number (RIN), Nanodrop spectrophotometer (Thermo Fisher Scientific, Waltham, MA, USA) for A260/A280 and A260/A230. RNA was measured quantitatively by Qubit 2.0 Flurometer (Life Technologies, Carlsbad, CA, USA). Inverse transcription using a SMARTer^®^ PCR cDNA Synthesis Kit (Takara Bio Inc., Kyoto, Japan) according to the manufacture’s protocol. The sixteen samples (4 treatments × 2 tissues × 2 biological replicates) were sequenced using Illumina HiSeq X 10 platform to generate pair-end reads with a length of 2 × 180 bp. The PacBio library construction, SMRT sequencing and data analysis for pooled RNA of sixteen individual samples were conducted to generate long reads. The cDNA was synthesized using PCR and amplicons were separated by agarose gel-based size selection into cDNA fractions of length 0.5–1 kb, 1–2 kb, 2–3 kb and 3–6 kb for four SMRTbell libraries construction. Sequencing was conducted by Nextomics Biosciences Co., Ltd (Wuhan, China). Subreads were filtered and subjected to self-correction via circular-consensus using the SMRT Analysis Server 2.2.0 (Pacific Biosciences of California, Inc, Menlo Park, CA, USA). Additionally, highly accurate NGS reads were used to correct SMRT sequence by the method in Au et al. [55].

Differential expression analysis between the four N conditions was performed using the DESeq2 [56]. The resulting P values were adjusted using the Benjamini and Hochberg approach to control the false discovery rate [57]. The criteria of corrected *p*-value < 0.005, log_2_ (Fold change) ≥ 1 and FDR (False Discovery Rate) < 0.001, were set as the threshold for significantly differential expression. To reduce transcription noise, each unigene was included for analysis only if its FPKM value was >0.01, a value chosen based on gene coverage saturation analysis (Table S4).

### 4.3. Verification of RNA-seq Data by qRT-PCR

Ten genes with different expression patterns revealed by RNA-seq were selected for validation by quantitative real-time PCR (qRT-PCR). RNA samples extracted from the roots and young leaves of the two independent biological replicates for each N treatment were employed for qRT-PCR validation. First-strand cDNA was synthesized using TRUEscript RT Master Mix (Aidlab biotechlonogies, Beijing, China). Primers designed for each gene are listed in Table S5. The expression levels of ribosomal protein gene *CsRPL13* in the samples were highly uniform and thus it was chosen as the internal control. The qRT-PCR analysis was conducted in triplicate as technical replicates using 2 × SYBR qRT-PCR Mix (Aidlab biotechnologies) using the StepOne Plusreal-time PCR system (Applied Biosystems, Waltham, MA, USA). Relative transcript levels were calculated using the comparative Ct method [58]. A regression analysis was performed between qRT-PCR and RNA-seq including all genes of four N treatments using the SAS package (version 9.2, http://www.sas.com).

### 4.4. Functional Annotation for Transcriptome Unigenes

For functional annotation, the expressed unigenes were annotated against the five databases of Nr, Swiss-prot, COG, KEGG and GO by BLASTX searches with an *e*-value < 1 × 10^−5^. Moreover, the isoforms were annotated against TPIA.

### 4.5. CsCAT9.1 Cloning and Transformation in Arabidopsis

T-DNA insertion *Arabidopsis* mutants provided by the Salk Institute Genomic Analysis Laboratory were ordered at the ABRC. The presence of the T-DNA insertion of SALK_042841 (exon, CAT9) was confirmed by sequencing PCR products with pROK2-specific left border primer (ATTTTGCCGATTTCGGAAC); all primers used are presented in Table S6.

The coding sequence of *CsCAT9.1* was amplified with primers presented in Table S6. The PCR amplicon was cloned into pTOPO-Blunt Simple vector (Aidlab Biotechnologies Co., Ltd.) and sequenced for sequence confirmation, then was digested and ligated into vector pBinGlyRed3 by restriction enzymes of *EcoR*I and *Xho*I. Then the recombinant plasmid was transformed into the *Agrobacterium tumefaciens* strain GV3101. Transformation of *Arabidopsis* (Col-0 and mutant SALK_042841) was performed via the floral dip method. Three transgenic independent homozygous lines (T_3_) were employed for AA uptake experiments.

### 4.6. Subcellular Localization and Amino Acid Substrates Identification of CsCAT9.1

The CDS of TEA020444/*CsCAT9.1* was fused to the N-terminus of the green fluorescent protein (GFP) in pMDC83 plasmid, and then the plasmid was transformed in tobacco (*Nicotiana benthamiana*) leaves for transient expression to determine the subcellular location. The plasmid carrying CaMV35S::GFP was used as an internal transformation control. To investigate *CsCAT9.1* subcellular localization, marker gene CBL1n and mCherry-HDEL [59], shown earlier to be localized in the plasma membrane (PM) and endoplasmic reticulum (ER), were used as internal controls. The transient expression method for subcellular location was described in Zhao et al. [60].

To understand the AAs substrates of *CsCAT9.1*, different *Arabidopsis* lines’ growth performances were determined and compared with individual AA added at different levels, and 3 mM NO_3_^−^ was used as a control of N source. Seeds of *Arabidopsis*, WT, mutant and T_3_ homozygous overexpression (OE) lines in WT or mutant background, were surface-sterilized and grown on Murashige and Skoog (MS) agar medium with a day/night photoperiod of 16/8 h at 24 °C/22 °C in square petri dishes (13 × 13 cm), after stratification of the seeds at 4 °C for 48 h. Three petri dishes represented three parallel biological repeats, and each line consisted of four seedlings in each dish. The number of rosette leaves per plant, fresh weight of whole plant (g), and primary root length (cm) were determined and averaged, each consisting of the tissues of four plants. Data were presented as mean ± standard deviation. OE1, OE2, OE3 and OE4 represent independent overexpression lines with WT background, and OE5 and OE6 represent independent complementary lines with *cat9* mutant background. Comparisons between OE1-4 and WT, OE5-6 and the mutant were statistically analyzed using SPSS 19.0, with significance levels at *p* = 0.05.

## Figures and Tables

**Table 1:** The number and percentage of annotated unigenes against five databases.

Databases	Number of Unigenes	Percentage
NR	41,238	96.07%
SwissProt	35,362	82.38%
KEGG	18,264	42.55%
GO	22,983	53.54%
KOG	26,841	62.53%
Annotated in all databases	10,615	24.73%
Annotated in at least one database	41,325	96.28%
Total unigenes	42,923	100%

## Supplementary Materials

The following are available online at https://www.mdpi.com/2223-7747/9/9/1218/s1, Figure S1: Length distribution of isoform level sequences. Figure S2: Length distribution of gene level sequences. Figure S3: Species distribution based protein sequences against NR database. Figure S4: GO enrichment classification for genes. Figure S5: KOG Function classification for genes. Figure S6: KEGG Function classification for genes. Figure S7: Expression levels of 28 CsNRT1, two CsNRT2 and five CsAMTs genes in shoots and in roots across the N treatments. Figure S8: Transcript levels of AAT genes in shoots and in roots across the N treatments. Figure S9: Expression levels of key genes involved in N assimilation in shoots and in roots across the N treatments. Figure S10: The NO3− treatment served as a control of N source for amino acid substrates identification. Figure S11: Main N transporters supposed to mediate key transportation processes. ‘√’ represents the potential candidates and ‘?’ means less possibility. Table S1: General properties of the reads produced by Pacbio RSII sequencing. Table S2: Confirmation of RNA-seq expression files with qRT-PCR. Table S3: The work solution formula for tea plant culture. Table S4: Differentially expressed genes identified by DESeq2. Table S5: Primers sequences for qRT-PCR analysis. Table S6: Primers sequence for Arabidopsis homozygous lines identification and CsCAT9.1 cloning.
