# Supplementary material for: Transcriptome-Wide Analysis of Nitrogen-Regulated Genes in Tea Plant (Camellia sinensis L. O. Kuntze) and Characterization of Amino Acid Transporter CsCAT9.1
Source: Plants (Basel). 2020 Sep 17;9(9):1218. doi: 10.3390/plants9091218 (PMC7569990; doi:10.3390/plants9091218)
Supplement: Supplementary file 1 [file plants-09-01218-s001.zip › plants-912709-supple-0/20200810Supplementary materials/Supplementary Figure S1-11/supplementary Figure S10.pptx]

## Slide 1
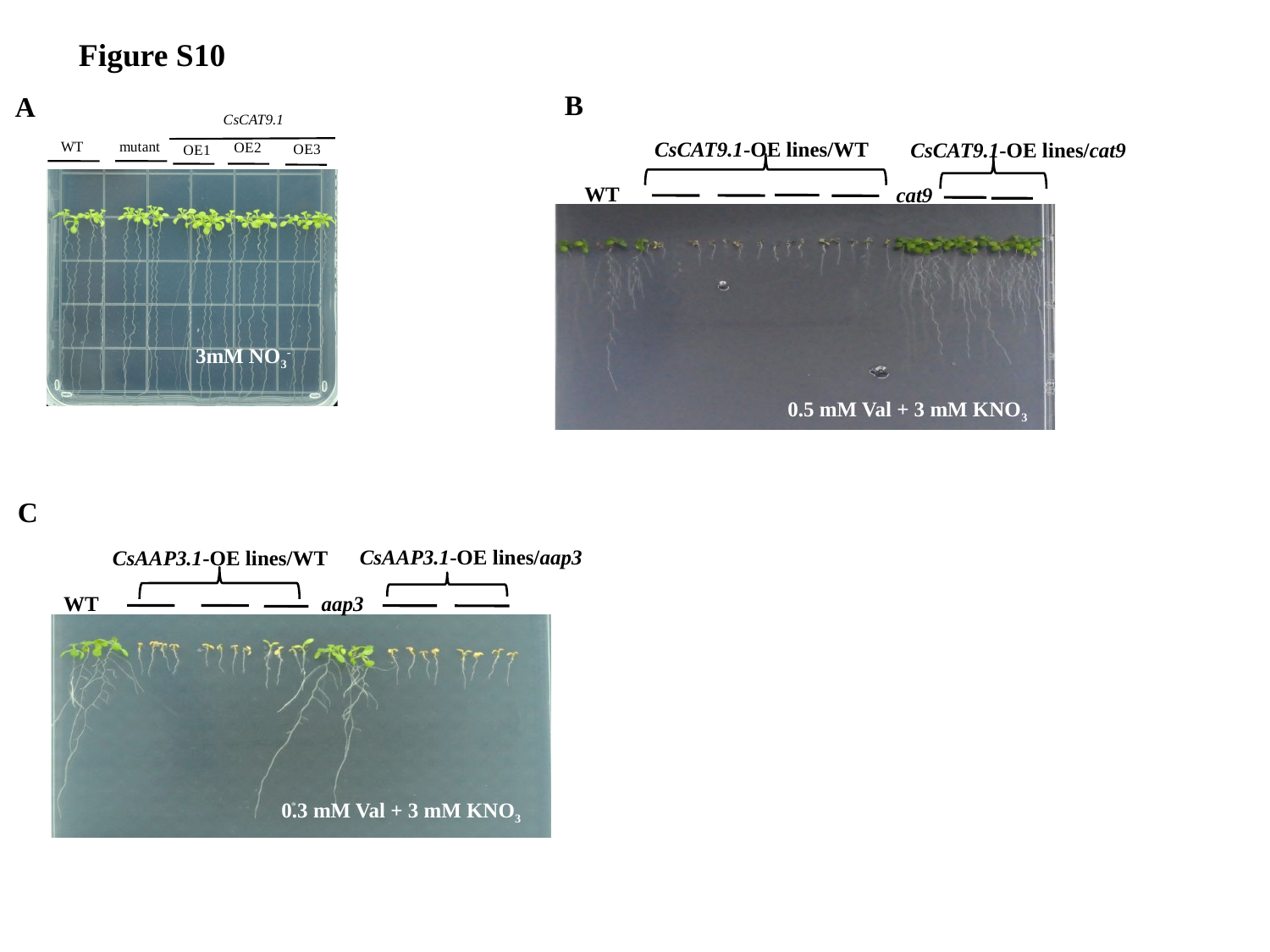

Figure S10
B
A
CsCAT9.1-OE lines/WT
CsCAT9.1-OE lines/cat9
WT
cat9
0.5 mM Val + 3 mM KNO3
3mM NO3-
C
CsAAP3.1-OE lines/aap3
CsAAP3.1-OE lines/WT
aap3
WT
0.3 mM Val + 3 mM KNO3
