# Supplementary material for: Transcriptome-Wide Analysis of Nitrogen-Regulated Genes in Tea Plant (Camellia sinensis L. O. Kuntze) and Characterization of Amino Acid Transporter CsCAT9.1
Source: Plants (Basel). 2020 Sep 17;9(9):1218. doi: 10.3390/plants9091218 (PMC7569990; doi:10.3390/plants9091218)
Supplement: Supplementary file 1 [file plants-09-01218-s001.zip › plants-912709-supple-0/20200810Supplementary materials/Supplementary Figure S1-11/Supplementary Figure S7 .pptx]

## Slide 1
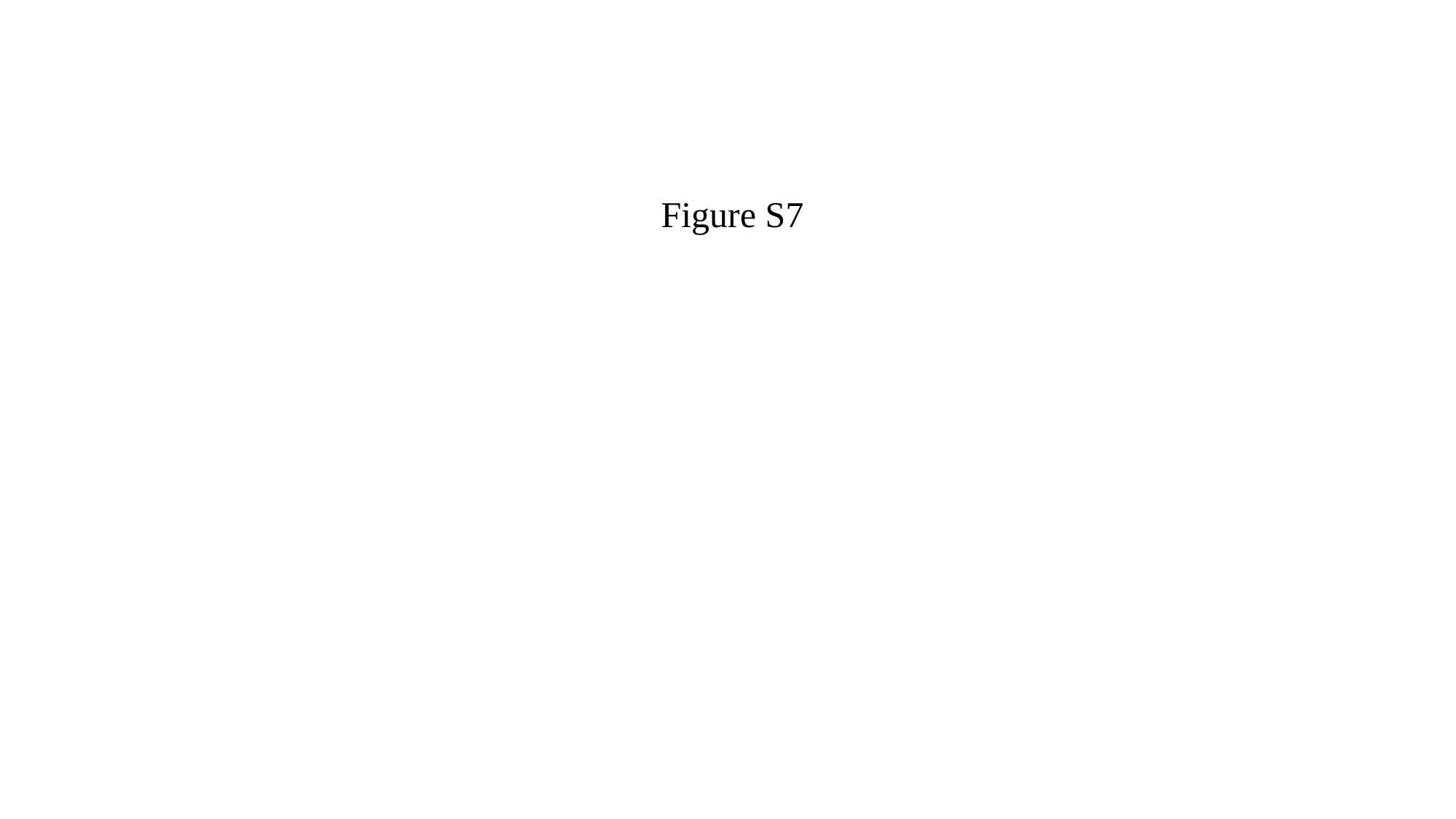

Figure S7

## Slide 2
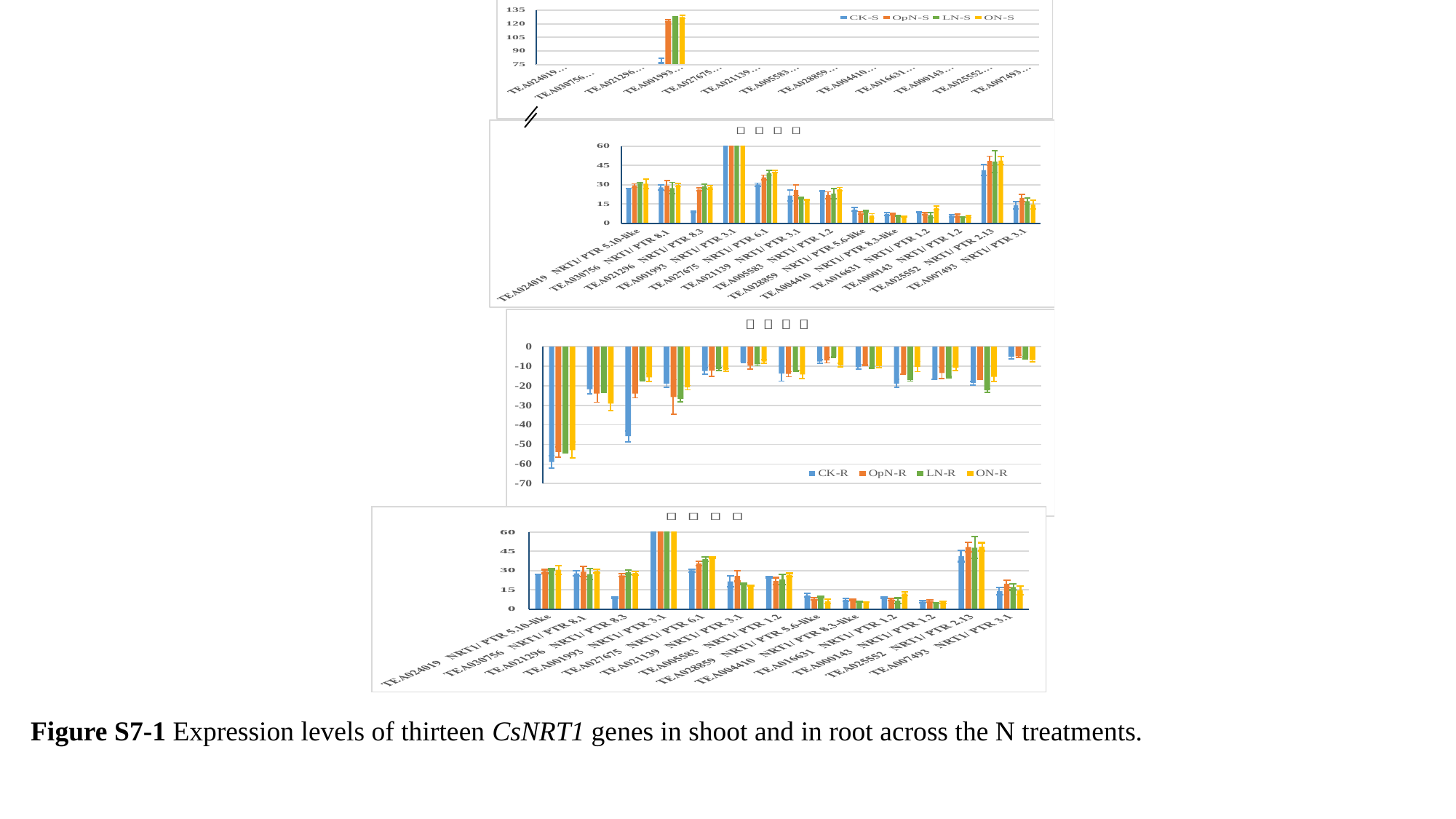

Figure S7-1 Expression levels of thirteen CsNRT1 genes in shoot and in root across the N treatments.

## Slide 3
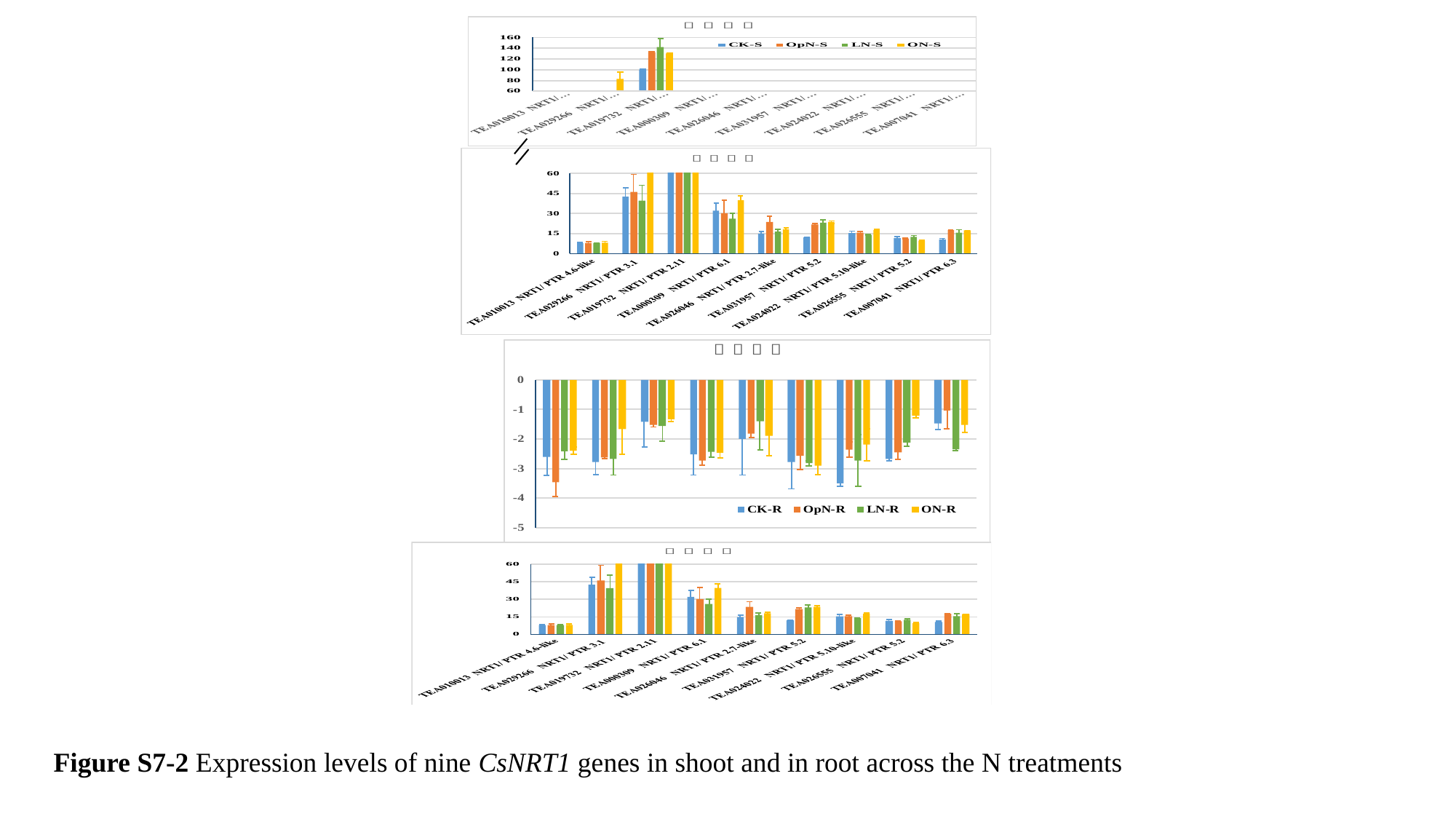

Figure S7-2 Expression levels of nine CsNRT1 genes in shoot and in root across the N treatments

## Slide 4
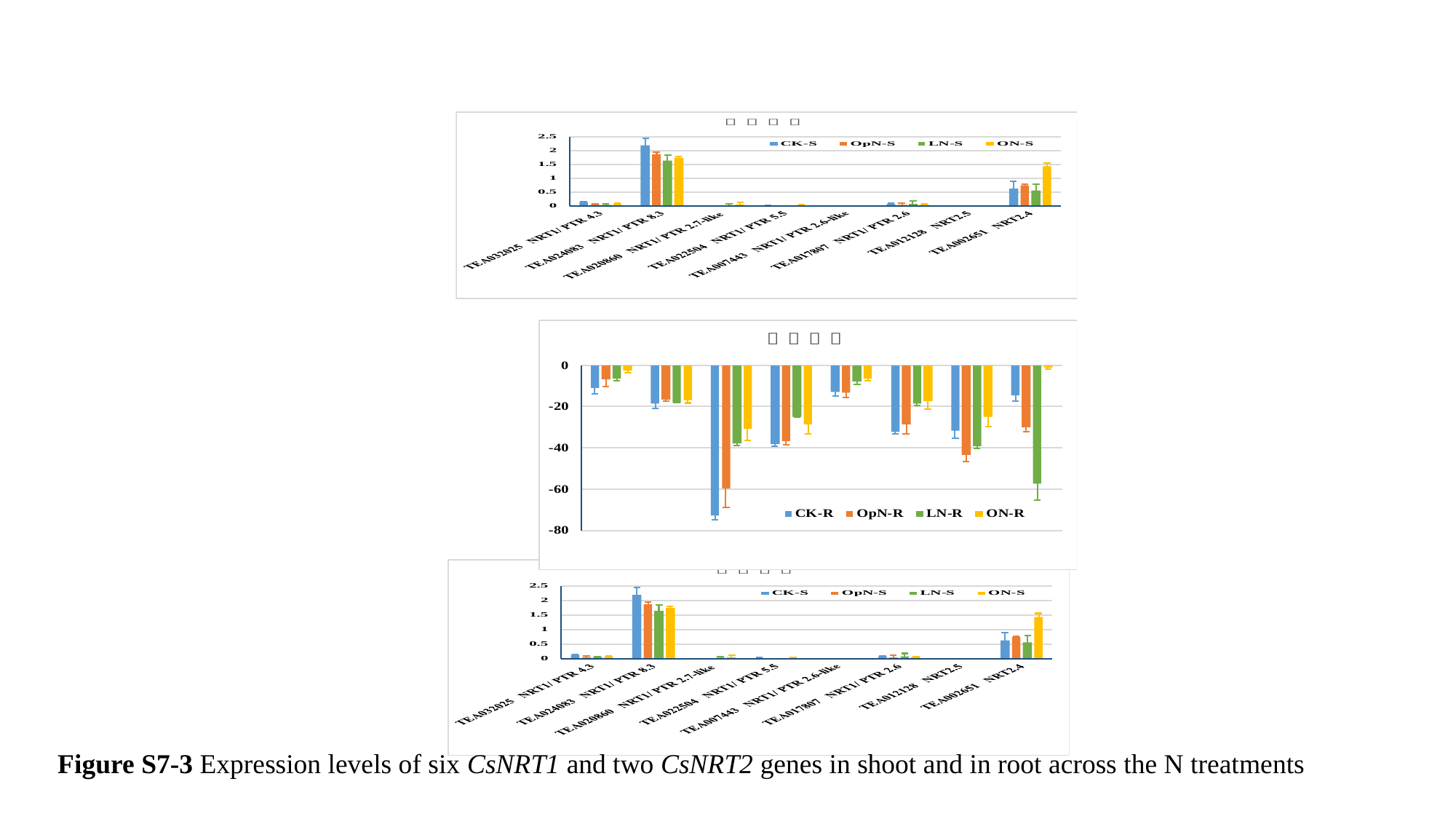

Figure S7-3 Expression levels of six CsNRT1 and two CsNRT2 genes in shoot and in root across the N treatments

## Slide 5
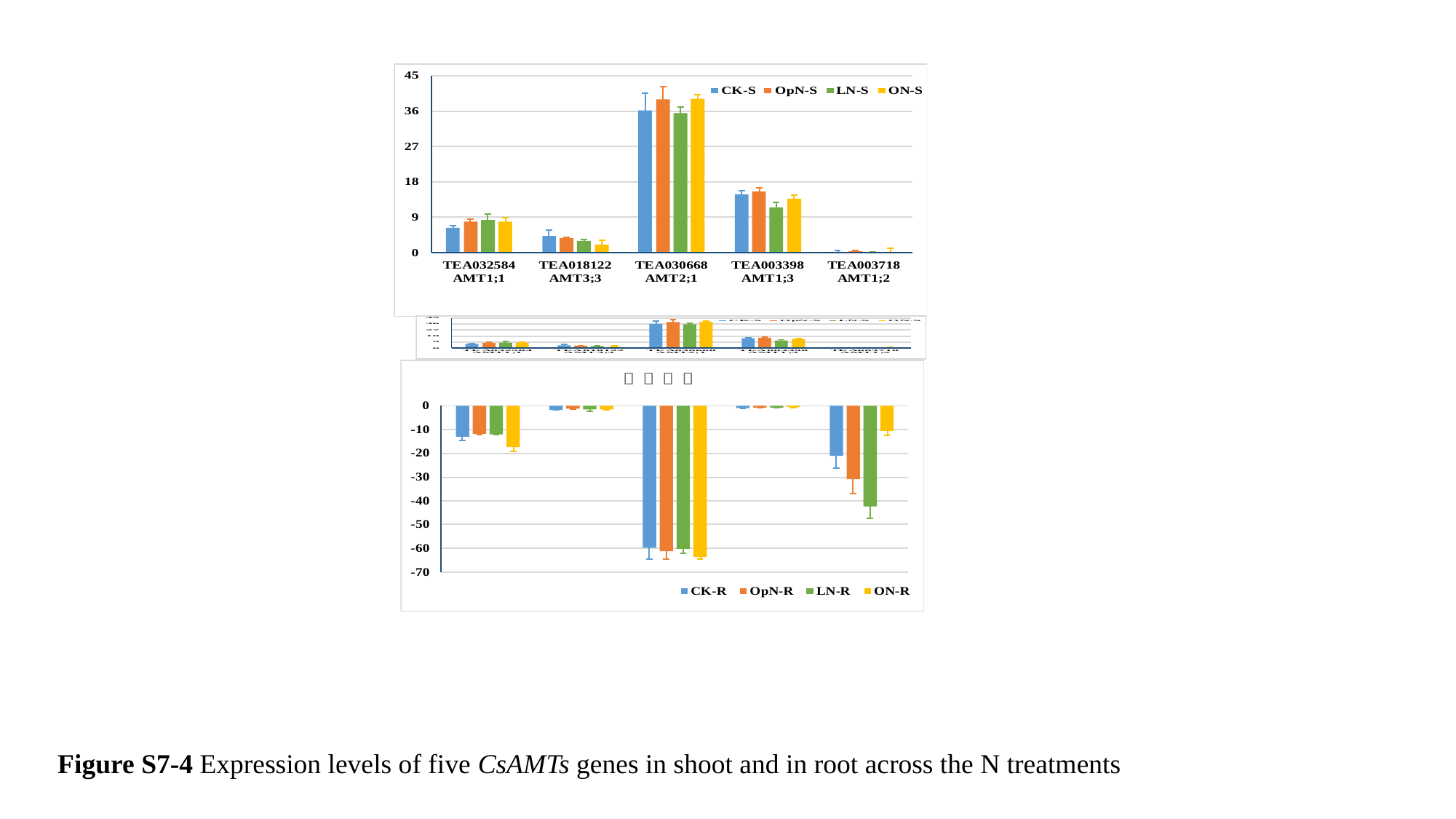

Figure S7-4 Expression levels of five CsAMTs genes in shoot and in root across the N treatments
