# Supplementary material for: Transcriptome-Wide Analysis of Nitrogen-Regulated Genes in Tea Plant (Camellia sinensis L. O. Kuntze) and Characterization of Amino Acid Transporter CsCAT9.1
Source: Plants (Basel). 2020 Sep 17;9(9):1218. doi: 10.3390/plants9091218 (PMC7569990; doi:10.3390/plants9091218)
Supplement: Supplementary file 1 [file plants-09-01218-s001.zip › plants-912709-supple-0/20200810Supplementary materials/Supplementary Figure S1-11/Supplementary Figure S8 .pptx]

## Slide 1
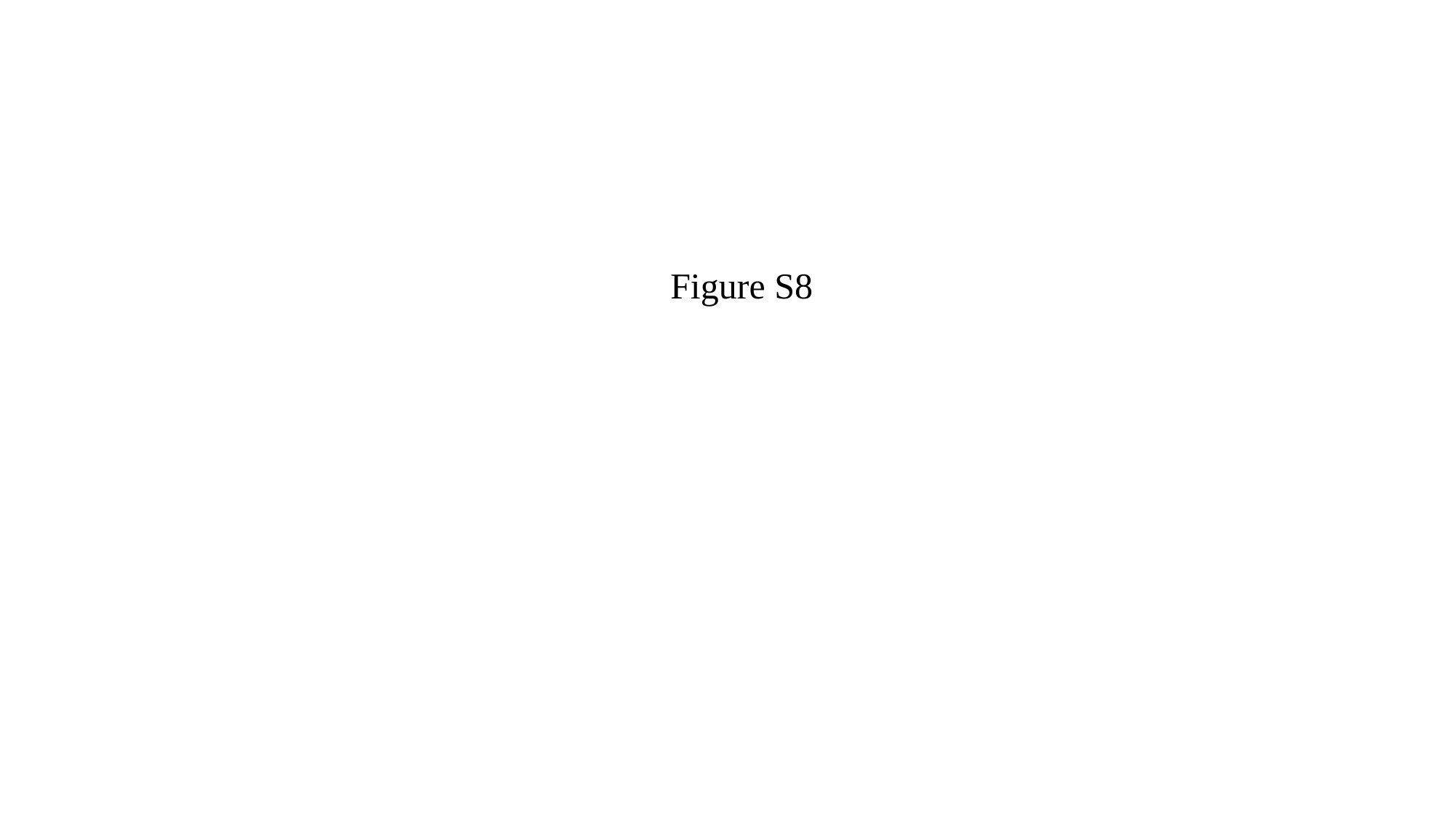

Figure S8

## Slide 2
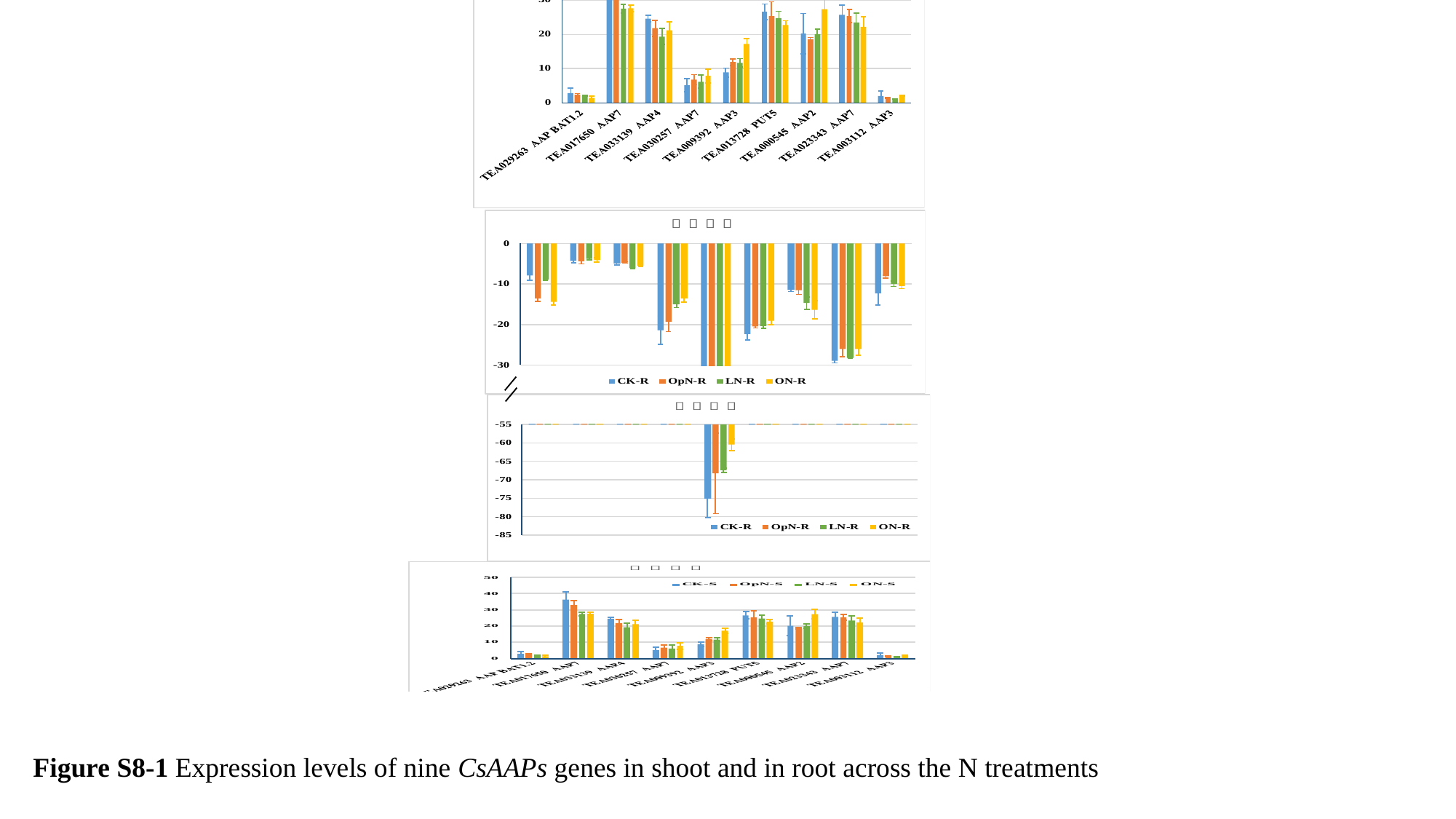

Figure S8-1 Expression levels of nine CsAAPs genes in shoot and in root across the N treatments

## Slide 3
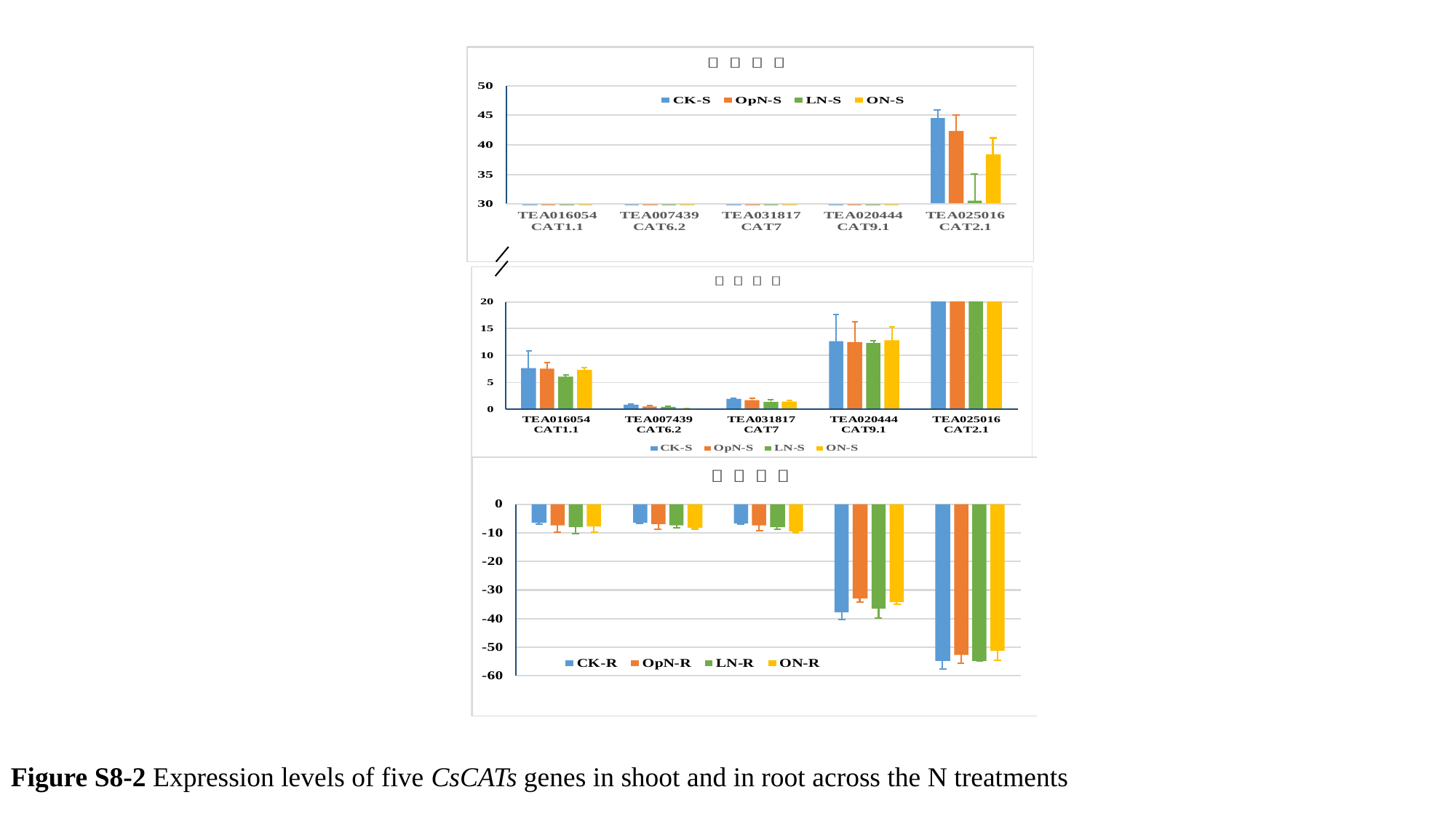

Figure S8-2 Expression levels of five CsCATs genes in shoot and in root across the N treatments

## Slide 4
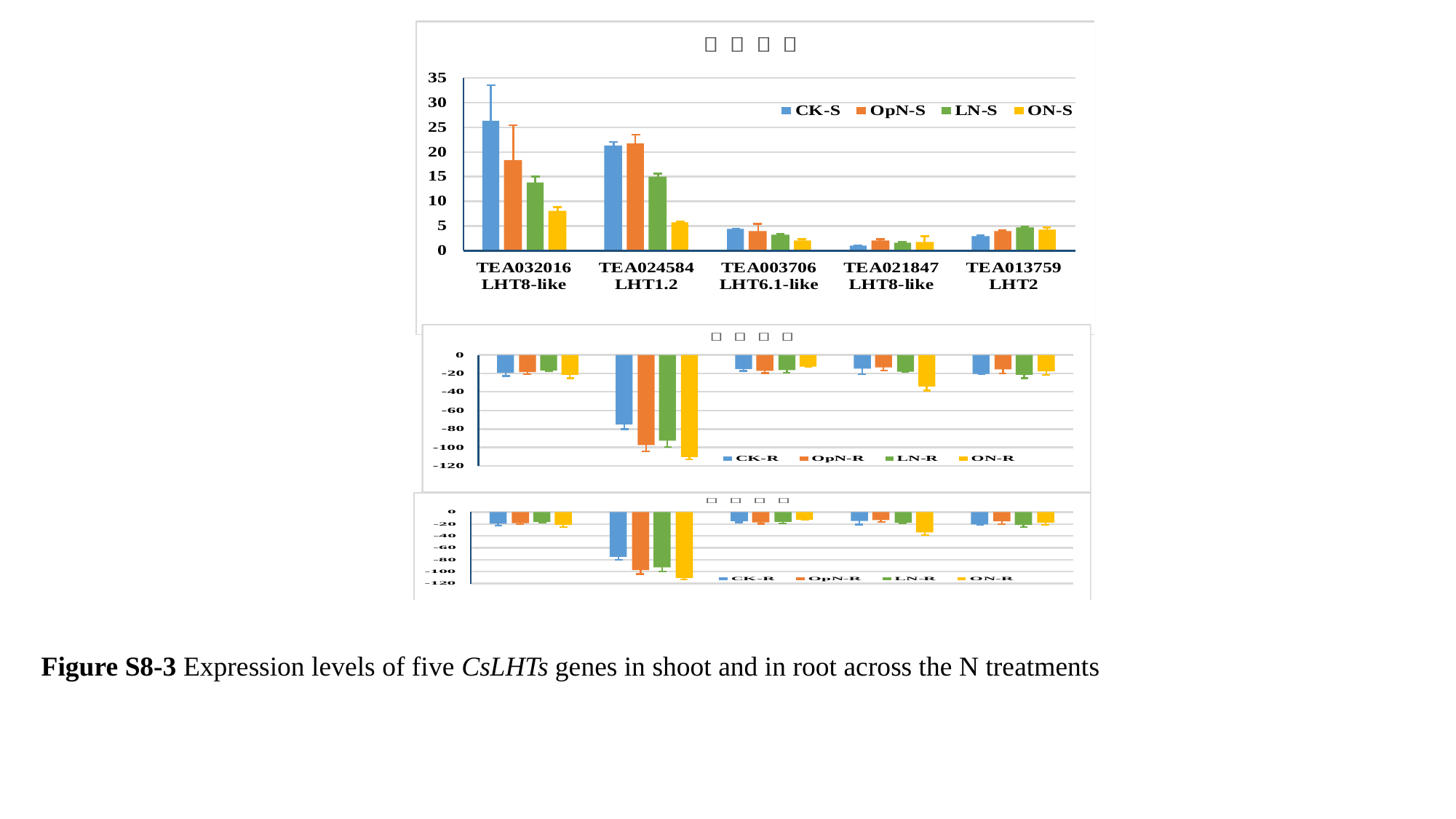

Figure S8-3 Expression levels of five CsLHTs genes in shoot and in root across the N treatments

## Slide 5
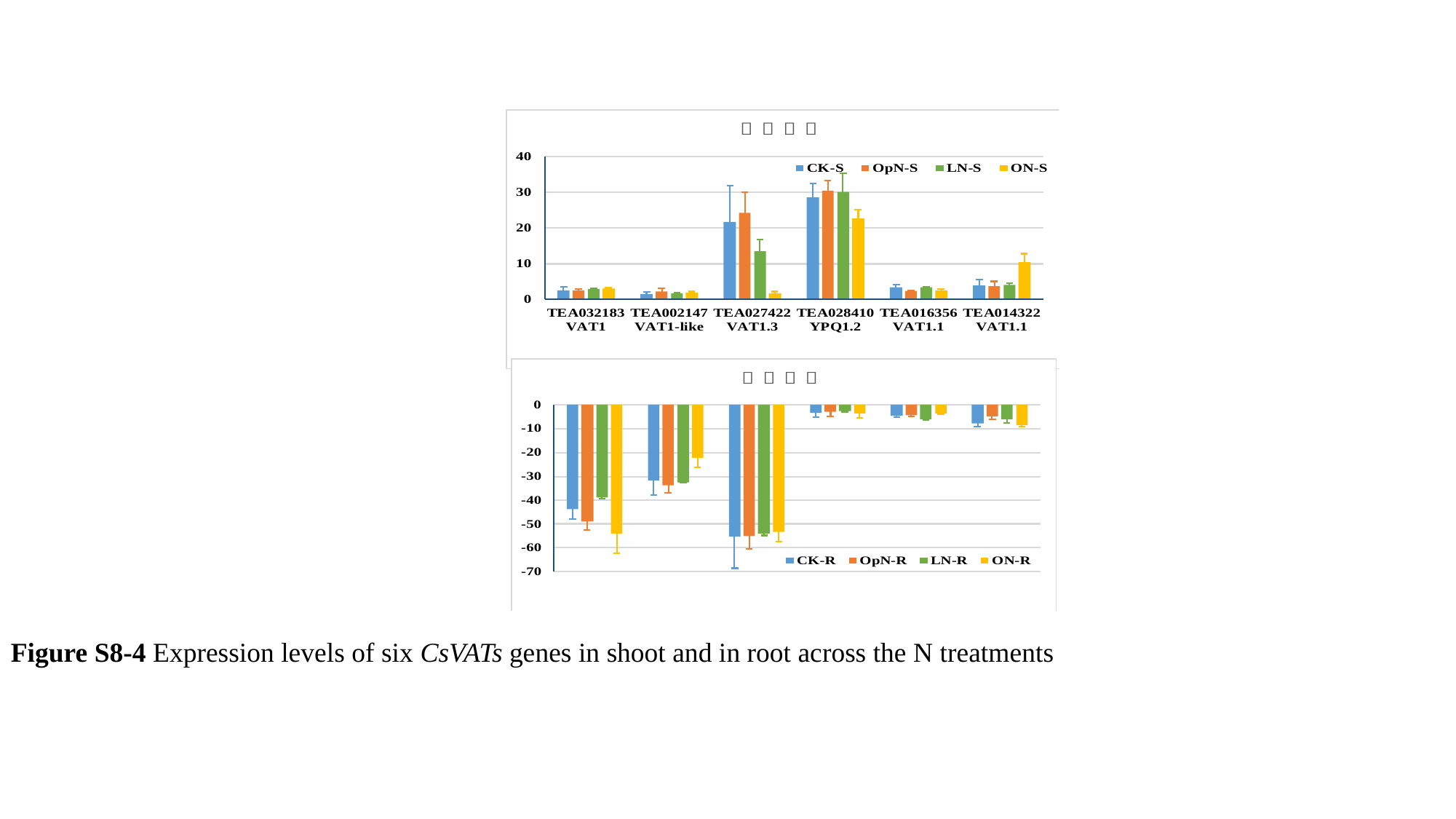

Figure S8-4 Expression levels of six CsVATs genes in shoot and in root across the N treatments

## Slide 6
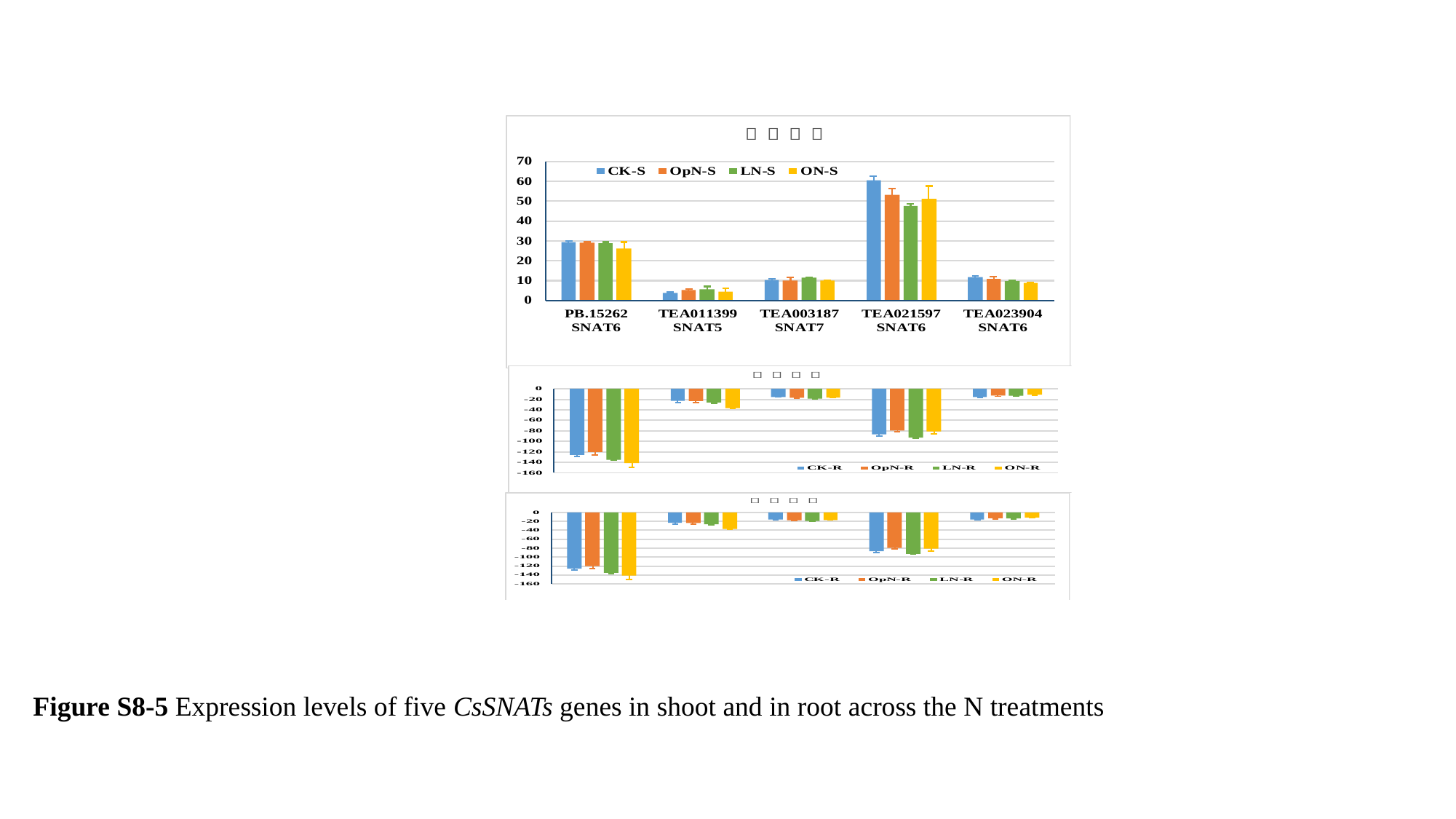

Figure S8-5 Expression levels of five CsSNATs genes in shoot and in root across the N treatments
