# Supplementary material for: Transcriptome-Wide Analysis of Nitrogen-Regulated Genes in Tea Plant (Camellia sinensis L. O. Kuntze) and Characterization of Amino Acid Transporter CsCAT9.1
Source: Plants (Basel). 2020 Sep 17;9(9):1218. doi: 10.3390/plants9091218 (PMC7569990; doi:10.3390/plants9091218)
Supplement: Supplementary file 1 [file plants-09-01218-s001.zip › plants-912709-supple-0/20200810Supplementary materials/Supplementary Table S1-5/Supplementary table S1 S3 S5 S6.docx]

**Table S1** General properties of the reads produced by Pacbio RSⅡ sequencing

| **Terms** | **0.5-1 kb** | | **1-2 kb** | | **2-3 kb** | | **> 3 kb** | |
| --- | --- | --- | --- | --- | --- | --- | --- | --- |
|  | **Value** | **Rate (%)** | **Value** | **Rate (%)** | **Value** | **Rate (%)** | **Value** | **Rate (%)** |
| #of reads of insert | 182,845 | 100 | 337,690 | 100 | 319,845 | 100 | 162,363 | 100 |
| #of five primer reads | 132,902 | 72.69 | 211,178 | 62.54 | 215,023 | 67.23 | 119，502 | 73.60 |
| # of three primer reads | 140,611 | 76.90 | 228,502 | 67.67 | 231,424 | 72.36 | 126,438 | 77.87 |
| # of poly-A reads | 139,988 | 76.56 | 227,720 | 67.43 | 232,373 | 72.65 | 126,707 | 78.04 |
| # of filtered short reads | 23,961 | 13.10 | 52,257 | 15.47 | 21,394 | 6.69 | 5,620 | 3.46 |
| # of non-full-length reads | 40,728 | 22.27 | 107,982 | 31.98 | 117,831 | 36.84 | 54,632 | 33.65 |
| # of full-length reads | 118,156 | 64.62 | 177,451 | 52.55 | 180,620 | 56.47 | 102,111 | 62.89 |
| # of full-length non-chimeric reads | 117,631 | 64.33 | 176,984 | 52.41 | 180,148 | 56.32 | 101,492 | 62.51 |
| Average full-length non-chimeric reads length | 1238 | - | 1765 | - | 2928 | - | 3705 | - |

**Table S3** The work solution formula for tea plant culture

|  |  | **Reagents** | **Work solution** | | |
| --- | --- | --- | --- | --- | --- |
|  |  |  | **CK/OpN** | **LN** | **ON** |
| Macronutrients | N | NH_4_NO_3_ | 0.83 mM | 0.083 mM | / |
|  |  | (NH_4_)_2_SO_4_ | 0.42 mM | 0.042 mM | / |
|  |  | Ca(NO_3_)_2_ 7H_2_O | 0.42 mM | 0.042 mM | / |
|  |  | Glu/Gly/His/Val/Thea | / | / | 30/30/30/30/30 μM |
|  | P | KH_2_PO_4_ | 1 mM | 1 mM | 1 mM |
|  | K | K_2_SO_4_ | 0.4 mM | 0.4 mM | 0.4 mM |
|  | Ca | CaCl_2_ 2H_2_O | 0.1 mM | 0.1 mM | 0.1 mM |
|  |  | CaSO_4_ | / | 0.38 mM | 0.38 mM |
|  | Mg | MgSO_4_·7H_2_O | 1.6 mM | 1.6 mM | 1.6 mM |
|  |  |  |  |  |  |
| Micronutrients | Fe | FeSO_4_ 7H_2_O | 35 μM | 35 μM | 35 μM |
|  |  | Na_2_EDTA | 35 μM | 35 μM | 35 μM |
|  | B | H_3_BO_3_ | 46.1 μM | 46.1 μM | 46.1 μM |
|  | Mn | MnSO_4_·H_2_O | 2.0 μM | 2.0 μM | 2.0 μM |
|  | Zn | ZnSO_4_·7H_2_O | 2.0 μM | 2.0 μM | 2.0 μM |
|  | Cu | CuSO_4_·5H_2_O | 0.3 μM | 0.3 μM | 0.3 μM |
|  | Mo | Na_2_MoO_4_·2H_2_O | 0.5 μM | 0.5 μM | 0.5 μM |
| Beneficial ele | Al | Al_2_(SO_4_)_3_·18H_2_O | 0.1 mM | 0.1 mM | 0.1 mM |

**Table S5** Primers sequences for qRT-PCR analysis

| **Code** | **Primer name.** | **Primer sequence (5’—3’)** | **Annealing temperature (℃)** |
| --- | --- | --- | --- |
| **1** | TEA027422-F | TTGCCGACCCAGTTCTA | 62 |
|  | TEA027422-R | GGTGGCGTATTGCTCCT |  |
| **2** | TEA013227-F | GGCTGGTATCGGTCGTG | 62 |
|  | TEA013227-R | TCCCAATCCTCCCTCCT |  |
| **3** | TEA000784-F | TCCGAGTAAAGACGAATGTG | 62 |
|  | TEA000784-R | GAGGTGGTGTTTGTGAAGG |  |
| **4** | TEA002651-F | AAATCCGACATAGGAAACG | 62 |
|  | TEA002651-R | CATCCAATACTGGCACGA |  |
| **5** | TEA003718-F | GTTGTTCTCGGCTCGTTC | 63 |
|  | TEA003718-R | AGGTAGTAAGTGCGGCTGT |  |
| **6** | TEA001683--F | TGTGAACCCGATTTATGGA | 62 |
|  | TEA001683--R | CTTGACAACAGCACCTGACTT |  |
| **7** | TEA032217--F | GGTGGCTCGTTACATCC | 62 |
|  | TEA032217-R | CGCTCGTTGCCTTCA |  |
| **8** | TEA026779-F | AGTTGTGCCAGCATCTAA | 62 |
|  | TEA026779-R | GACGTGACTATCTTCTCCC |  |
| **9** | TEA009696-F | GGCAAAGGACATAGAAGTG | 62 |
|  | TEA009696-R | CAGTAAACGAGGACGAAGA |  |
| **10** | TEA002643-F | AGAGGCGTTCAAGGTGT | 62 |
|  | TEA002643-R | AAGCCAAAGCAGCAAA |  |
| **11** | CsRPL13-F | GCTCAAGGCAGCAGGTAT | 62 |
|  | CsRPL13-R | TGGCTTCTCACGCACAA |  |

**Table S6** Primers sequence for Arabidopsis homozygous line identification and gene cloning

| **Gene name** | **Primers’ name** | **Primers’ sequence（5’-3’）** | **Tm value（℃）** | **T-DNA insert site** |
| --- | --- | --- | --- | --- |
| Primers for T-DNA insertion site confirmation | | | | |
| *CsCAT9.1* | SALKseq_042841-LP | TCGTTCTCGTTGTGATTTGTG | 52.5 | exon |
|  | SALKseq_042841-RP | TGAAACGGTAAAATACGCCAG |  |  |
|  | TDNA-specific | ATTTTGCCGATTTCGGAAC |  |  |
| Primers for gene cloning | | | | |
| *CsCAT9.1* | *CsCAT9.1F*-EcoRI-F | TAGAATTCATGGGGGGAGAGAAGAGC | 55 | / |
|  | *CsCAT9.1R*-XhoI-R | GCCTCGAGTTATTGAGTTTCTTCTTCAGG |  |  |
