# Supplementary figures and images for: Transcriptome-Wide Analysis of Nitrogen-Regulated Genes in Tea Plant (Camellia sinensis L. O. Kuntze) and Characterization of Amino Acid Transporter CsCAT9.1
Source: Plants (Basel). 2020 Sep 17;9(9):1218. doi: 10.3390/plants9091218 (PMC7569990; doi:10.3390/plants9091218)

Histogram for Length Distribution

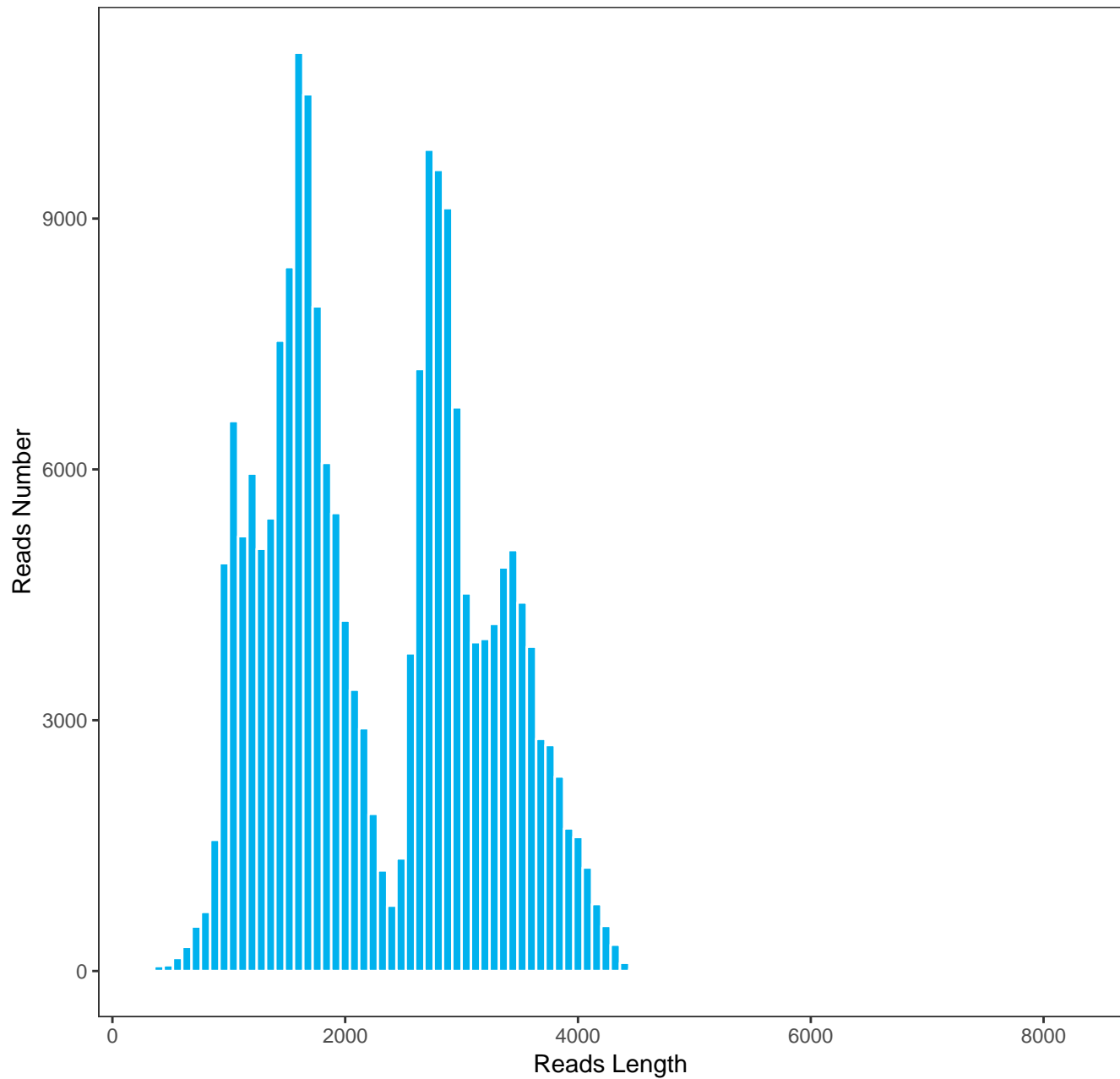

Supplement: Supplementary file 1 [file plants-09-01218-s001.zip › plants-912709-supple-0/20200810Supplementary materials/Supplementary Figure S1-11/Supplementary Figure S1.pdf]

Histogram for Length Distribution

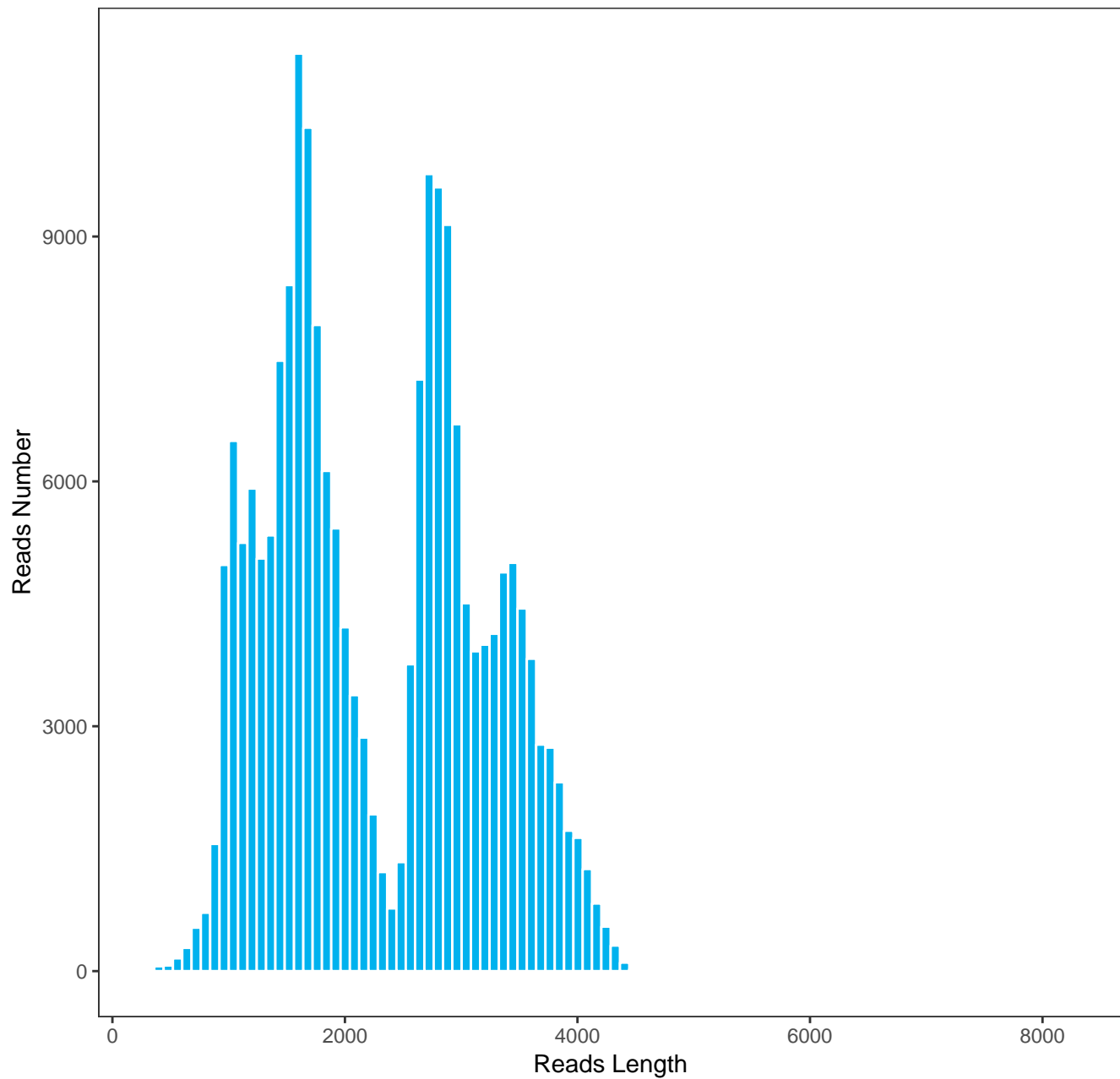

Supplement: Supplementary file 1 [file plants-09-01218-s001.zip › plants-912709-supple-0/20200810Supplementary materials/Supplementary Figure S1-11/Supplementary Figure S2.pdf]

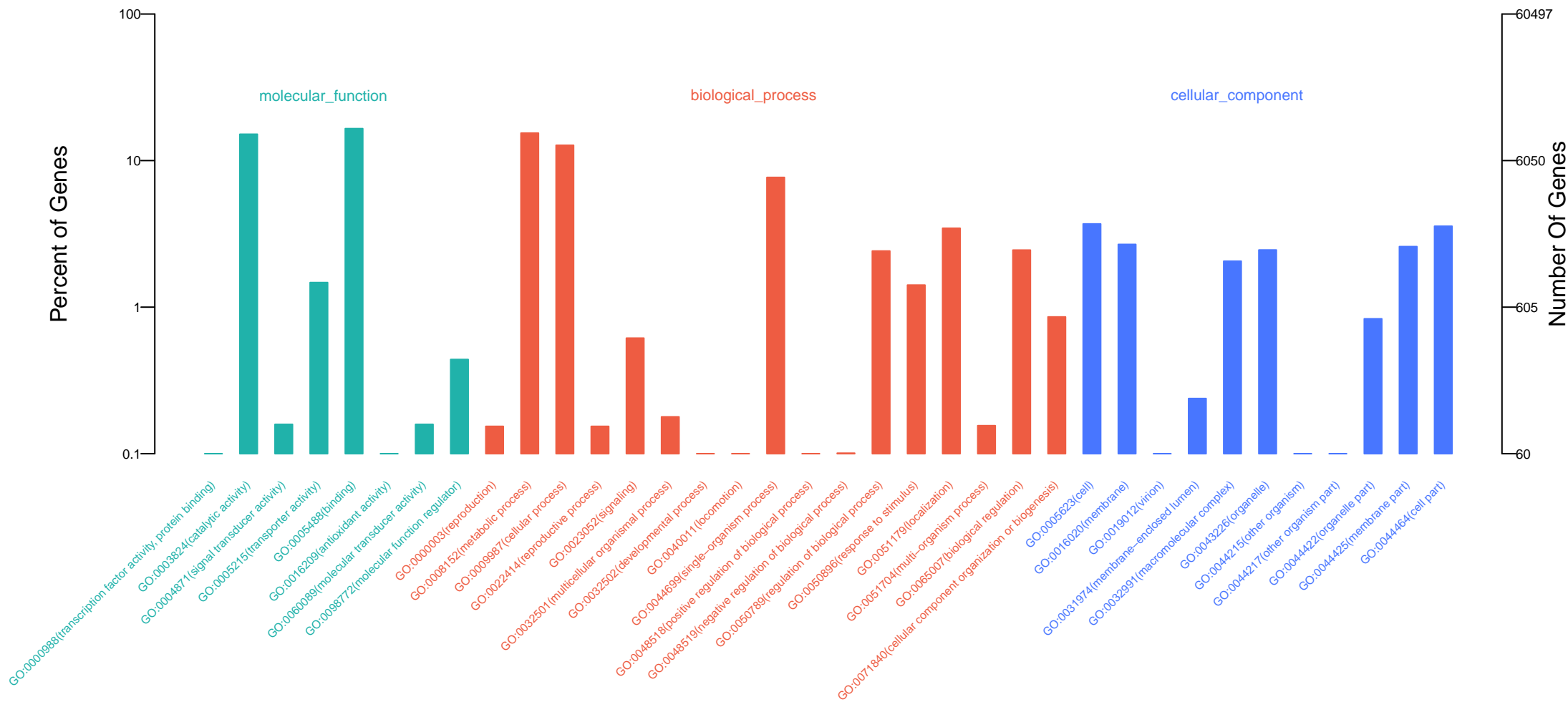

Supplement: Supplementary file 1 [file plants-09-01218-s001.zip › plants-912709-supple-0/20200810Supplementary materials/Supplementary Figure S1-11/Supplementary Figure S4.pdf]

KOG Function Classification

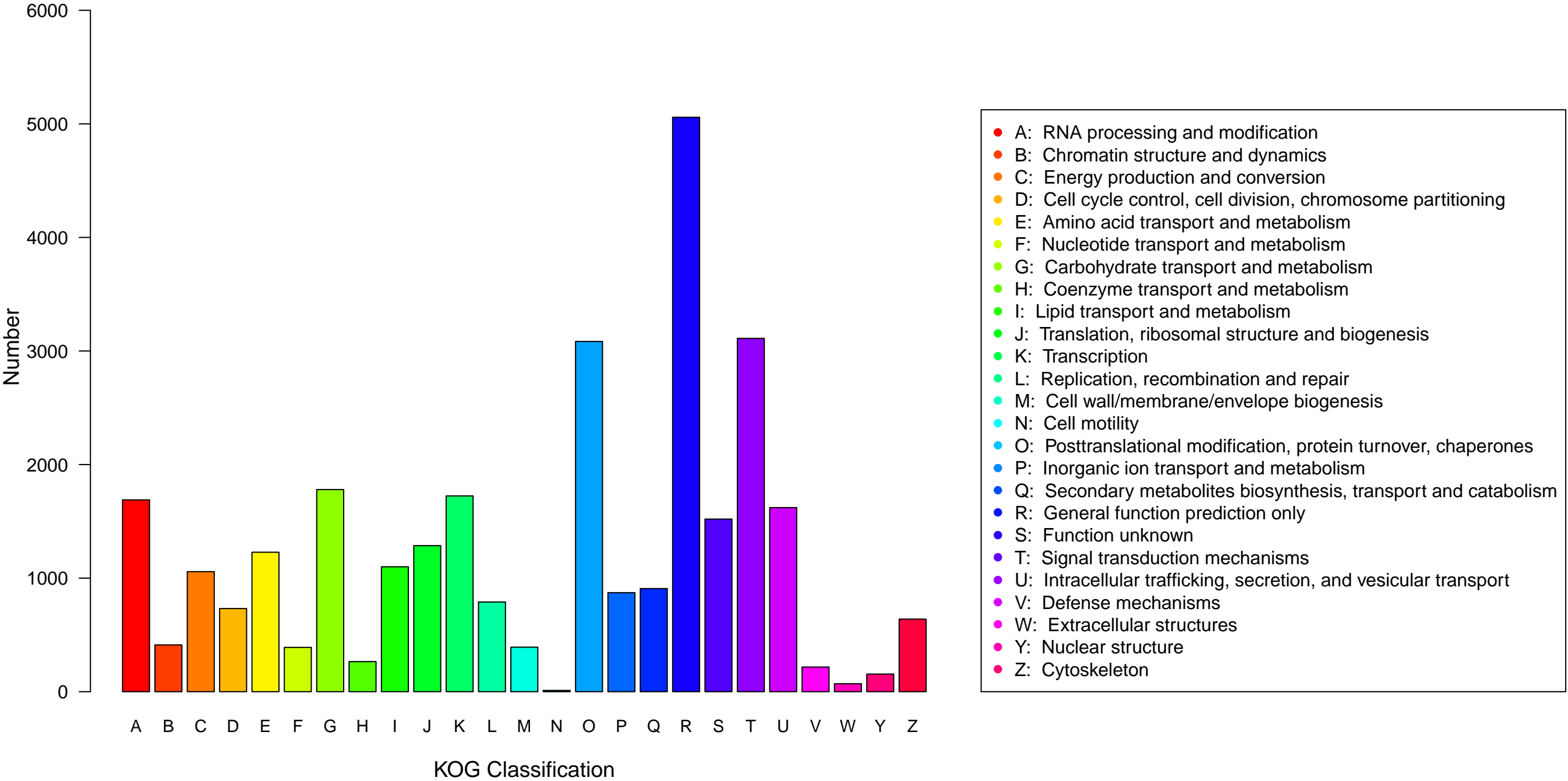

Supplement: Supplementary file 1 [file plants-09-01218-s001.zip › plants-912709-supple-0/20200810Supplementary materials/Supplementary Figure S1-11/Supplementary Figure S5.pdf]

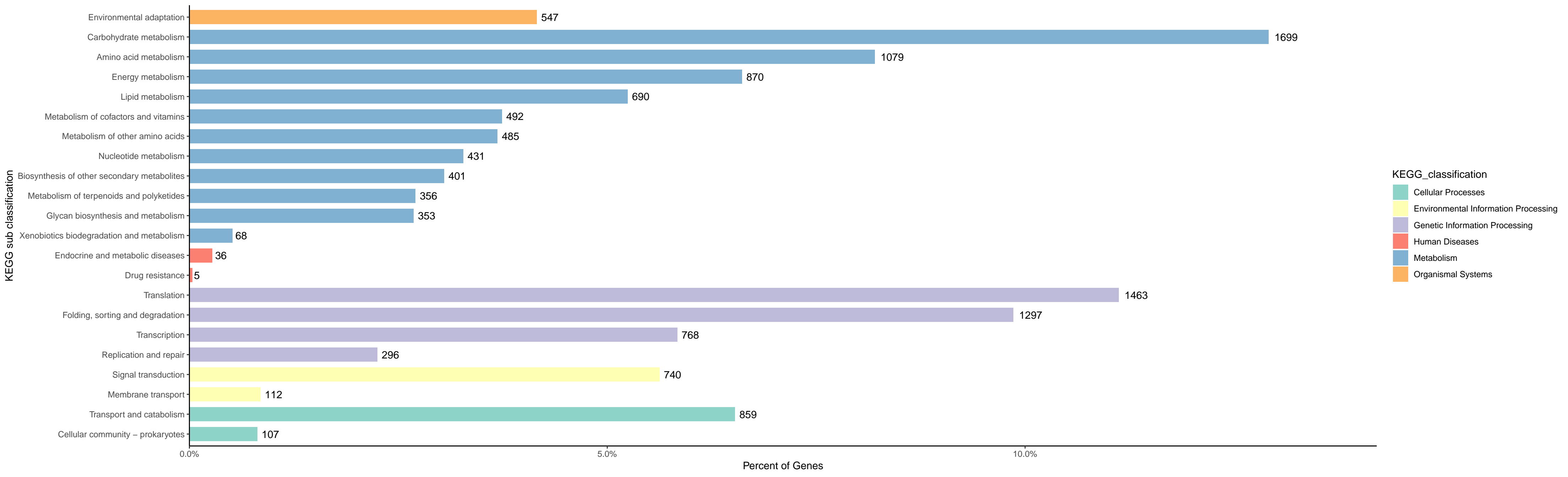

Supplement: Supplementary file 1 [file plants-09-01218-s001.zip › plants-912709-supple-0/20200810Supplementary materials/Supplementary Figure S1-11/Supplementary Figure S6.pdf]
